# Deep learning for simultaneous phase and amplitude identification in coherent beam combination

**DOI:** 10.1038/s41598-025-96385-w

**Published:** 2025-04-06

**Authors:** Fedor Chernikov, Yunhui Xie, James A. Grant-Jacob, Yuchen Liu, Michalis N. Zervas, Ben Mills

**Affiliations:** Optoelectronics Research Centre, University of Southampton, Southampton, UK

**Keywords:** Applied optics, Optics and photonics, Optical techniques, Imaging and sensing

## Abstract

Coherent beam combination has emerged as a promising strategy for overcoming the power limitations of individual fibre lasers. This approach relies on maintaining precise phase difference between the constituent beamlets, which are typically established using phase retrieval algorithms. However, phase locking is often studied under the assumption that the power levels of the beamlets remain stable, an idealisation that does not hold always in practical applications. Over the operational lifetime of fibre lasers, power degradation inevitably occurs, introducing additional challenges to phase retrieval. To address this, we propose a deep learning algorithm for single-step simultaneous phase and amplitude identification, directly from a single camera observation of the intensity distribution of the combined beam. By leveraging its ability to detect and interpret subtle variations in intensity interference patterns, the deep learning approach can accurately disentangle phase and power contributions, even in the presence of significant power fluctuations. Using a spatial light modulator, we systematically investigate the impact of power-level fluctuations on phase retrieval within a simulated coherent beam combination system. Furthermore, we explore the scalability of this deep learning approach by evaluating its ability to achieve the required phase and amplitude precision as the number of beamlets increases.

**Supplementary Information:**

The online version contains supplementary material available at 10.1038/s41598-025-96385-w.

## Introduction

Over the past few decades, substantial advancements have been made in power scaling of high-power fibre lasers (HPFLs)^1–3^, with stable operation at kilowatt-level average powers being demonstrated across a wide range of wavelengths in single-mode fibre systems. However, as power levels increase, several nonlinear effects, such as stimulated Raman scattering^4^, stimulated Brillouin scattering^5^, and optical Kerr effect^6^, become more pronounced, severely limiting further scaling. A common strategy to mitigate these, often intensity-dependent, nonlinearities is to increase the fibre core size, thereby reducing the intensity across the core area. Nonetheless, increasing core size introduces new challenges such that large-core fibres tend to support multiple modes, and thermo-optically induced effects, such as power coupling between modes (e.g., transverse mode instability^7^), make achieving stable single-mode operation highly challenging. To overcome these fundamental limitations, alternative approaches have been explored, as a promising solution to the power scaling challenges faced by HPFLs.

Coherent Beam Combination (CBC)^8–11^ aims to combine the amplified outputs of multiple HPFLs in phase, operating each below the pumping threshold that would otherwise induce optical nonlinearities and instability, to achieve a total power output beyond what is typically achievable in a single-mode HPFL. A key engineering challenge in CBC is maintaining mutual coherence between the fibre outputs, especially given the varying phase noise introduced by the parallel amplified stages. In addition to power scaling, CBC enables other advanced functionalities, such as non-mechanical beam steering^12^ and the generation of exotic beam profiles^13^. These capabilities require not only the suppression of phase noise but also precise and accurate control over the relative phases between the fibre outputs. Developing a reliable, high-performance, and cost-effective phase-locking system is therefore essential to the success of CBC.

A straightforward approach to infer relative phase information, denoted as $\:\varphi\:$, from intensity distribution observations, denoted as $\:I$, is to approximate the inverse mapping function from intensity observations to corresponding relative phase information (i.e., approximating $\:f:I\to\:\varphi\:$). Whilst the analytical expression that maps phase information to intensity distributions (i.e., $\:\varphi\:\mapsto\:I$) is well-documented in the literature^14^, the inverse mapping $\:f:I\to\:\varphi\:$ typically requires numerical and/or iterative methods to solve. This necessity arises from the nature of the inverse problem inherent in intensity calculations due to their quadratic dependence on the optical field modulus, thus rendering analytical solutions generally infeasible. Previous endeavours have employed phase retrieval approaches that measure spatial interference or beating patterns^15,16^, which often necessitates additional hardware such as reference beams or beam samplers. Other iterative phase retrieval approaches have also been explored, including the Gerchberg-Saxton algorithm, hybrid input-output (HIO)^17^, hill-climbing, stochastic parallel gradient descent (SPGD)^18^, and reinforcement learning^19–22^.

Recent studies demonstrate that this inverse mapping $\:f:I\to\:\varphi\:$ can be effectively approximated using convolutional neural networks^23,24^ (hereafter the singular is referred to as NN), which enables single-step inference of relative phases from intensity distributions of a beamlet array, under the assumption that each comprising fibre is operating at a constant power level. From a practical perspective, however, laser output power can decrease over its operational lifetime. Photodarkening, for instance, can reduce output power by as much as 20%^25^. Complete failure of one of the channels is also possible. In this work, we build out previous efforts in single-step phase inference from far-field intensity distributions using NNs^26^. We extend this approach to also infer relative amplitude variations between the fibre outputs, further enhancing the capabilities of the CBC systems. Additionally, we explore phase inference under various possible power degradation levels, providing insights into the robustness of the NN approach in practical scenarios. Beyond expanding the inference capabilities of NNs, we address the critical question of how the number of training pairs required to achieve predetermined phase and amplitude precision scales with the number of beamlets. This scalability analysis provides valuable insights into the practicality of using the NN approach for CBC systems with a higher number of beamlets.

## Methods

### Experimental setup

**Fig. 1:**
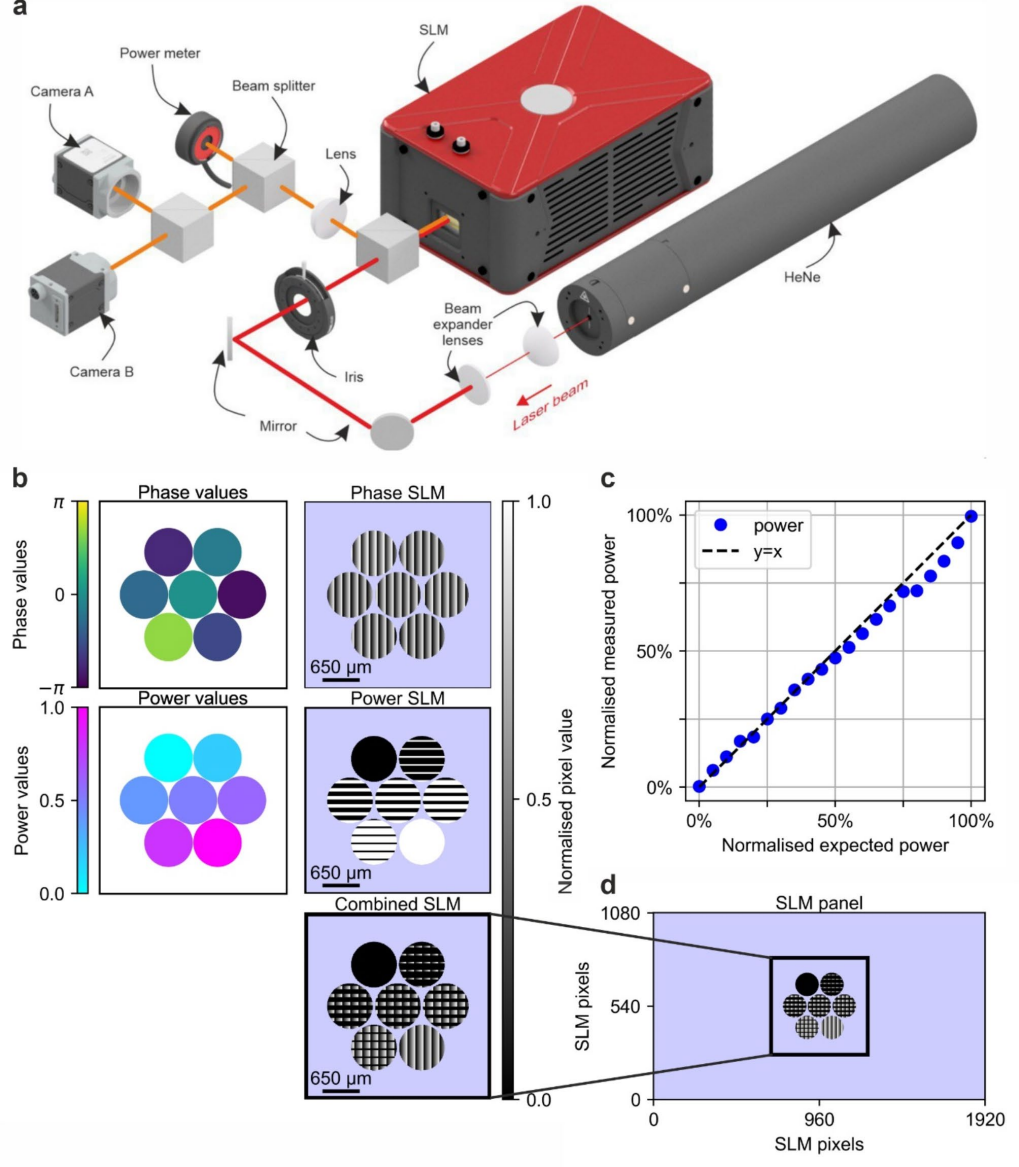
(**a**) Schematic of the experimental setup (**b**) Independent encoding of phase and amplitude information onto each composing beamlet, where phase modulation is applied by a blazed grating pattern and amplitude modulation is applied by a binary grating pattern. The combined phase and amplitude modulations are superimposed to form the pattern displayed on the SLM, as shown in (**d**) (**c**) Accuracy of power control for individual beamlet.

A Gaussian-profiled, continuous-wave, linearly polarised, and intensity-stabilised beam from a Helium-Neon laser source (Thorlabs, HRS015B, 632.992 nm central wavelength, 1.2 mW output power) was expanded and collimated, before being directed onto a Liquid-Crystal-on-Silicon Spatial Light Modulator (Thorlabs EXULUS-HD1/M, 1920 × 1080 pixel resolution, 6.5 μm pitch size, hereafter referred to as the SLM) at normal incidence via a non-polarising 50:50 cube beam splitter (Thorlabs CCM1-BS013/M). The beam was expanded to the maximum size permissible by the optical elements, and an iris was positioned before the SLM to ensure that only portion of the beam within a restricted aperture was transmitted. The iris truncated the Gaussian profile of the beam, allowing transmission of the central part and thereby transforming the profile of the beam from Gaussian to quasi-top-hat. The modulated beam, reflected from the SLM, was redirected by the same beam splitter towards a convex lens (focal length 40 cm) and subsequently passed through a cascade of two 50:50 beam splitters (Thorlabs CCM1-BS013/M). These split the focused light into three parts: the first beam, immediately after the initial beam splitter, was directed to a photodiode power sensor (Thorlabs S120C), whilst the second and third beams, split by the second beam splitter, were directed to two cameras (Basler a2A4504-18umBAS). Specifically, a camera (hereafter referred to as Camera A) was positioned approximately 10 cm before the focal plane, capturing the intensity distribution at that plane, whereas the other camera (hereafter referred to as Camera B) was positioned exactly at the focal plane to capture the far-field intensity distribution. A schematic of the experimental setup is shown in the Fig. 1a.

To simulate collimated outputs from a centrosymmetric, close-packed array of optical fibres, a pattern was displayed on the active area of the SLM, programmatically assigning both relative phase and amplitude to each simulated fibre output. The relative phase and amplitude were encoded for each simulated fibre output using two overlapping, circularly shaped grating patterns, as shown in Fig. 1b, with Fig. 1d illustrating their implementation on the SLM panel. Specifically, a blazed pattern in the horizontal direction, where the phase distribution modulates horizontally and cycles linearly from $\:-\pi\:$ to $\:+\pi\:$, encoded the phase information into higher-order diffraction outputs in the horizontal direction by setting the initial phase values. In this configuration, if two simulated fibres shared the same phase cycling periodicity, they would interfere in-phase at the far field if their initial phase values were identical, or out-of-phase if their initial phase values differed by $\:\pi\:$. The relative amplitude was encoded by a binary grating pattern in the vertical direction, where the phase distribution modulates in a periodic binary manner. This binary grating consisted of alternating series of “teeth”: one series with a uniform phase value of 0, and the other series using the phase values from the horizontal blazed grating. The ratio between these two types of teeth determined the relative intensities observed at higher-order diffraction outputs in the horizontal direction at the far field. That is, the binary grating created additional diffraction orders in the vertical direction for each horizontal diffraction order, redistributing the intensity vertically based on the periodicity of the binary grating. To confirm that the power modulation behaved as expected, the combined power was measured using a photodiode power sensor placed specifically in the diffraction order of main interest and plotted against the expected power. The resulting linear dependence, as shown in Fig. 1c, confirms the expected relationship between the applied and measured power. Both the initial phase value of the blazed grating and the periodicity of the binary grating can be independently adjusted for each simulated fibre, allowing independent control of their relative phases and intensities, thereby effectively controlling both the relative phases and relative amplitudes of the simulated fibres.

### Data collection

In this work, the mapping $\:f:I\to\:(P,\varphi\:)$ is approximated by a function approximator $\:F$ parameterised by $\:\theta\:$ (i.e., NN), and is trained on a dataset comprising intensity observations $\:I=\{{I}_{0},{I}_{1},\dots\:\}$ (i.e., each of shape $\:n\times\:n$) and their corresponding relative amplitude levels $\:P=\{{P}_{0},{P}_{1},\dots\:\}$ and phases $\:\varphi\:=\left\{{\varphi\:}_{0},{\varphi\:}_{1},\dots\:\right\}$ (i.e., ground truth). The objective is to identify a set of parameters $\:\theta\:$ that minimises an objective function $\:J(F\left(I;\theta\:\right),P,\varphi\:)$, which quantifies the discrepancy between the predicted relative phases and amplitudes $\:F(I;\theta\:)$ and their respective ground truth $\:\{P,\varphi\:\}$, commonly known as supervised learning. Notably, whilst this learning can be conceptually viewed as parameterising a mapping: $\:f:{\mathbb{R}}^{n\times\:n}\to\:\mathbb{C}$, for practical reasons we relax the mapping to $\:{:\mathbb{R}}^{n\times\:n}\to\:{\mathbb{R}}^{2}$. The inclusion of amplitude information is critical because intensity distributions are influenced not only by phase differences but also by amplitude variations of the interfering beamlets. It is generally known that in beam interference, relative phase differences alone result predominantly in interference “fringe” shifts, whilst relative amplitude variations alone result predominantly in “fringe” visibility changes. Whilst these dependencies are obvious in the two-beam case, in multibeam interference, i.e., the generalised sum of two-beam interference, they cannot be easily discerned. This underscores the importance of accounting for both amplitude and phase parameters in the mapping. Provided that the dataset $\:(I,P,\varphi\:)$ is of sufficient quality and quantity, the function approximator $\:F(I,\theta\:)$ typically generalises well, enabling it to approximate $\:f$ effectively. Nonetheless, this raises an important question: does this generalisation capacity extend robustly to cases involving power fluctuations, where varying amplitudes could potentially impact the performance of $\:F$? Investigating this robustness is particularly relevant for multibeam interference scenarios.

**Fig. 2:**
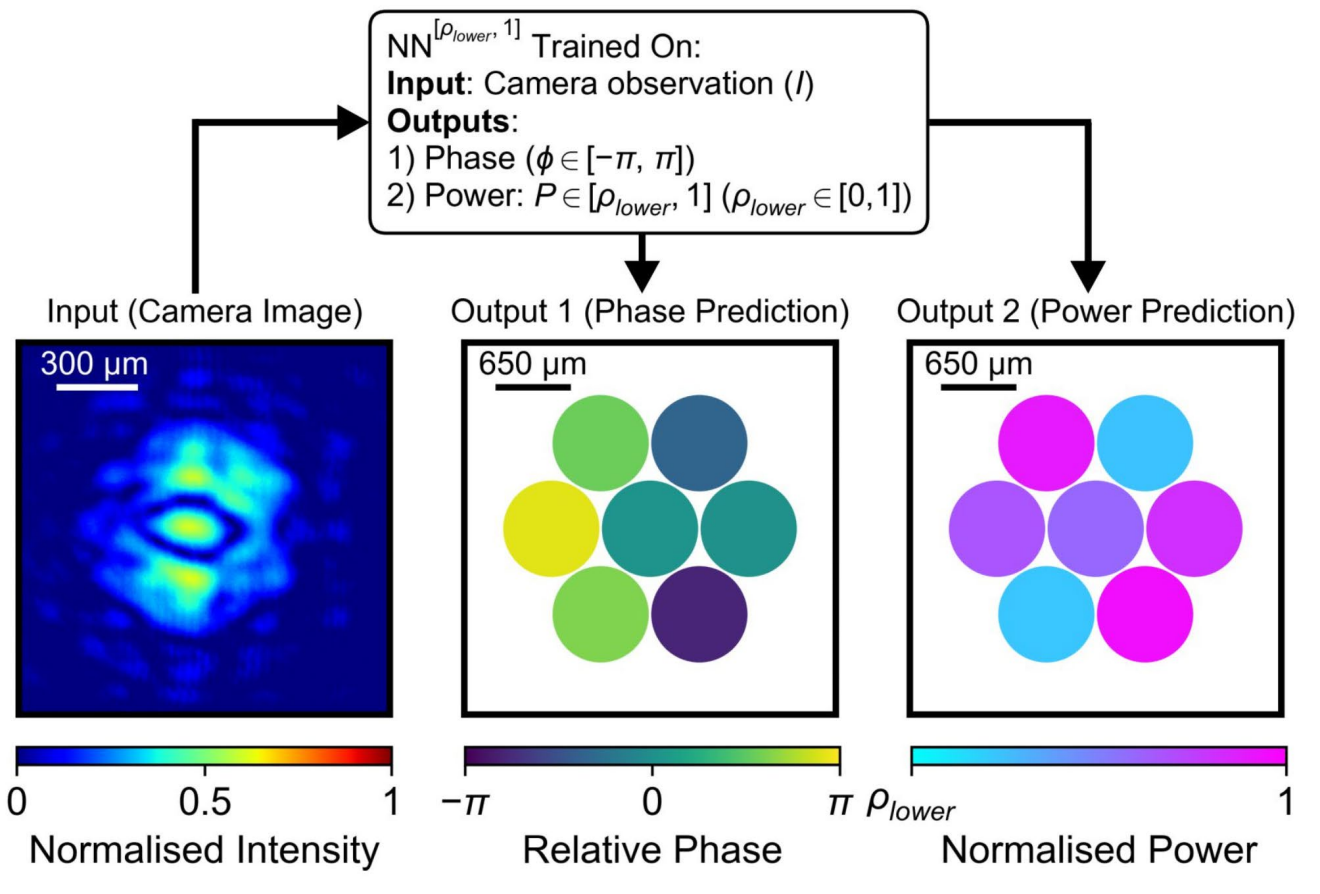
The neural networks considered in this study were trained on datasets consisting of training pairs where the input was the intensity distributions measured 10 cm before the focal plane, and the output comprised the corresponding phase and power values for each beamlet. These intensity distributions were generated by randomly assigning phase values within the range $\:\left[-\pi\:,\:\pi\:\right]$ and power values within the range $\:\left[{\rho\:}_{lower},\:1\right]$ ($\:{\rho\:}_{lower}\in\:\left[0,\:1\right]$, where 1 stands for 100% power), sampled from uniform distributions to each beamlet. Here, $\:{\rho\:}_{lower}$ represents the lower boundary of the power variation range, which was allowed to vary, whilst the upper boundary was fixed at 1. This enabled the assessment of the impact of power fluctuations on the phase extraction for CBC system.

To investigate the impact of power fluctuations on phase inference accuracy, training datasets were collected where both the relative phases and the power levels of composing beamlets were varying uniformly at random. Consequently, each training pair consisted of a camera observation of the intensity distribution (input to NN) and a corresponding ground truth that included two distinct physical quantities: phases (i.e., $\:\varphi\:$) and power levels (denoted as $\:P$), as shown in Fig. 2. By limiting the lower limit for power level variations, multiple training datasets were generated, enabling comparative ablation studies to assess the impact of power fluctuations on the phase information inferred from observations of focused beamlet array.

The training data were collected using a simulated 7-fibre hexagonal close-packed beamlet array with a geometric fill factor of approximately 74.9%. The phase values of the outer six beamlets were varied uniformly at random within the range between $\:-\pi\:$ and $\:+\pi\:$, with the phase of the beamlet at the centre fixed at $\:0\pi\:$ to serve as a reference beam. The relative phases of the surrounding beamlets were then inferred from their interference pattern at the far field after the lens (10 cm before the focal plane). The power levels of the individual outer beamlets varied uniformly at random within the range $\:\left[{\rho\:}_{lower},1\right]$, where $\:{\rho\:}_{lower}\in\:\left[\text{0,1}\right]$ is the lower boundary of the power variation range and the upper boundary was fixed at 1 (i.e., 100% of maximum power). This indicates that up to ($\:1-{\rho\:}_{lower})\times\:100\%$ of maximum power of the horizontal high-order diffraction outputs was redistributed vertically due to the applied binary grating. The power level of the central beamlet was not fixed and was allowed to vary within the same range $\:[{\rho\:}_{lower},1]$ as the outer beamlets, whilst its phase remained fixed at $\:0\pi\:$. A number of training datasets were collected by varying only the power range [$\:{\rho\:}_{lower}$, 1] whilst the phase variation range was consistently set between $\:[-\pi\:,\pi\:]$ for all datasets.

After the pattern that encoded phase and power information to higher-order diffraction outputs was displayed on the active area of the SLM with the corresponding set of phases and power levels, the combined intensity distribution was observed and captured from Camera A and Camera B simultaneously. Throughout this work, the intensity distributions captured from Camera A (positioned 10 cm before the focal plane) were used as the input to NN for inferring relative phase information, whilst Camera B (positioned at the focal plane) was utilised to observe the far-field intensity distributions at focus. Each training data pair was generated by randomly assigning phase values and varying power levels to composing beamlets, then capturing the corresponding intensity distributions from both cameras. The training pair comprised an intensity distribution from Camera A and the corresponding 6 phase values for the outer 6 beamlets (excluding the fixed phase of the central fibre) and 7 power values for all the composing beamlets.

**Fig. 3:**
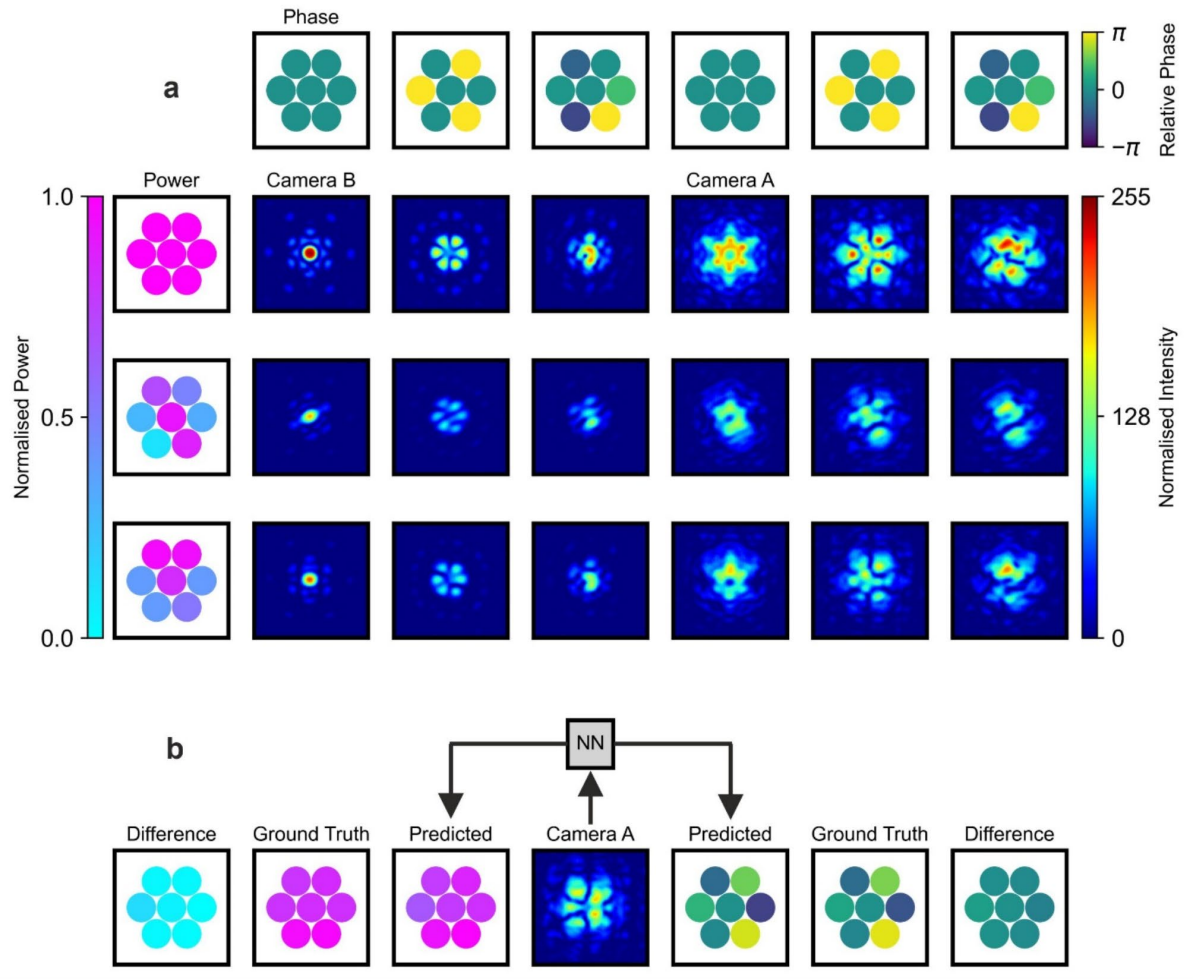
(**a**) Examples of far-field intensity patterns recorded on Camera B and intensity patterns recorded 10 cm before the focus on Camera A for different combinations of phase and power values. These patterns correspond to the same set of phases and powers but are recorded at different locations, resulting in a total of nine observations each for Camera A and Camera B. (**b**) Example of simultaneous NN predictions of the phases and powers of composing beamlets from a single Camera A observation. The results are displayed with the predicted values, ground truth values, and their differences arranged vertically, one under another, for clear comparison.

To demonstrate how phase and power alter the intensity distributions both in the far-field and 10 cm before the focus, Fig. 3a presents 9 examples of Camera B observations and 9 corresponding examples recorded by Camera A. In these examples, the horizontal rows show observations corresponding to fixed power values, where only the phase values assigned to each beamlet are varied. Each row represents a specific power profile, illustrating how the relative phase affects the overall shape of the combined beam whilst the power remains unchanged. Conversely, the vertical columns correspond to fixed phase values, where only the power values of the beamlets are varied. Changes in power along the columns further modify the combined beam shape, with the intensity distributions preserving the main features defined by the phase whilst altering the generalised “fringe” visibility in the interference patterns. Although both phase and power influence the combined intensity distribution, they do so in distinct ways, enabling the NN approach to extract both phase and power information from a single camera observation of the intensity distribution. Comparing patterns along rows and columns, it becomes obvious that phase-only variations have a more profound effect on interference than amplitude-only variations. This is more evident in the well-focused (camera B) patterns. It is important to note that whilst Camera A observations are recorded 10 cm before the focus to break Fourier symmetry and allow for phase extraction, the resulting interference patterns still combine contributions from all beamlets, meaning the individual beamlets are not directly visible. In a near-field setup, the power of each beamlet could have been measured directly with a single camera; however, in this case, the power is also inferred from the combined intensity distribution. This highlights the ability of the NN to accurately deduce both phase and power from interference patterns. Figure 3b demonstrates how the NN simultaneously predicts six phase and seven power values from a single Camera A observation. For clarity, the ground truth phase and power values are displayed alongside the differences between the predicted and ground truth values.

### Neural network

The NN architecture was based on MobileNetV3-Small with a width multiplier of 1.0, chosen for its computational efficiency and low latency. Whilst other architectures, such as ResNet^27^, exist and may potentially offer similar or better prediction accuracy and precision, MobileNetV3 was preferred due to its ability to achieve latencies in the hundreds of hertz (417.88 ± 88.56 Hz for MobileNetV3-Small, measured on a Windows 10 system with an Intel i7-7700 CPU @ 3.60 GHz, GPU Nvidia Quadro P6000), compared to the significantly lower speed of under 100 Hz (59.36 ± 13.53 Hz) for ResNet-18 (see Supplementary Material 3). However, since the MobileNetV3 architecture was originally designed for image classification task (i.e., mapping an input to a probability distribution of a given number of discrete classes), it cannot be directly used for inferring phase information from intensity distributions, which can be seen as a regression task (i.e., mapping an input to a continuous variable). Several necessary modifications were therefore made to its original design. Firstly, during data collection, the image from Camera A was cropped to 540 × 540 pixels to encompass the region containing the majority of non-zero pixel values of the interference pattern. This cropped image was subsequently down-sampled to 256 × 256 pixels using pixel area resampling. In the original design of MobileNetV3, the input image size was 224 × 224 pixels, however, for this work, it was adjusted to 256 × 256 pixels, which slightly increased the dimensions of the final feature maps from 7 × 7 to 8 × 8. Another key modification was made to the size of the output layer. The output was reduced from the original *N* × 1000 to *N* × 13 (i.e., 6 phase values and 7 power values). The final major change involved adjusting the loss function to account for the periodic nature of the phase. A trigonometric loss function was employed, taking the form: $\:J(\phi\:,\:\varphi\:)={\left(cos\phi\:-cos\varphi\:\right)}^{2}+{\left(sin\phi\:-sin\varphi\:\right)}^{2}$, where $\:\phi\:$ and $\:\varphi\:$ represent the predicted and ground truth phase values, respectively^23,26^. To include the prediction of varying power levels, an additional Mean Squared Error (MSE) term $\:{\left(\rho\:-P\right)}^{2}$, where $\:\rho\:$ and $\:P$ are predicted and ground truth power values, respectively, was incorporated into the loss function. The final loss function consisted of two terms, namely, the trigonometric loss term, used for predicting relative phases, and the MSE loss, used for predicting powers. Due to the differences in numerical scales of these terms, there was a risk of disproportionate influence on the overall loss, potentially compromising the prediction accuracy for phases and powers. To address this, a weighting factor was applied to the MSE loss term, reducing its magnitude by two orders of magnitude. This weighting factor ensured that the numerical scale of the MSE loss term was comparable to that of the trigonometric loss term, enabling the NN to focus approximately equally on predicting both phases and powers. Throughout this work, the NNs that were trained under different power variation ranges are denoted as $\:{NN}^{\left[{\rho\:}_{lower},1\right]}$, where $\:\left[{\rho\:}_{lower},1\right]$ specifies the range of the power variations included in the training dataset for each respective NN.

For NNs presented later in this work (unless stated otherwise), datasets containing 50,000 training pairs were experimentally collected and split with a ratio of 90:10 for training and validation. The training set was used to optimise the parameters $\:\theta\:$ in NNs (i.e., with backpropagation enabled), whilst the validation set was reserved for evaluating the performance of the NNs (i.e., with backpropagation disabled), providing an objective measure of the performance of NNs. All NNs were trained over 200 training epochs, iterating over the entire dataset during each epoch with the Adam optimiser (learning rate of $\:6e-5$). Upon completion of training, the final NN weights were saved for subsequent assessment.

## Results

### Neural network evaluation

In general, studies on phase inference in CBC research are conducted under the assumption that the power output of each comprising beamlet is stable. These studies, characterised by the absence of power fluctuations, represent an idealised case often explored in CBC research, where each channel maintains a stable power output over time. NNs that are trained on these fixed-power datasets often demonstrated accurate phase inference under these idealised conditions^26^. In practical applications, however, power degradation is commonly observed over the lifetime of a fibre amplifier. This raises two key questions. First, how would a NN trained under these idealised conditions perform over time as the fibres degrade and their power outputs fluctuate? Second, would incorporating power variations into the training dataset enhance the robustness and performance of the NN approach? To address these questions, we introduce an evaluation metric that incorporates datasets containing samples collected with varying power levels. Using this metric, we assess the performance of NNs that are trained using the datasets that are collected under different power variation conditions, on the same evaluation set. This approach allows us to simulate the potential decline in phase inference performance of NNs as the fibres age and their power outputs fluctuate, providing valuable insights into the robustness of the NN approach.

The evaluation datasets, which feature variations in both phase and power levels, were collected separately from the training and validation datasets to ensure they contained data not seen by NNs during training. The only distinction among these datasets was the degree of power variation. This enabled the assessment of NNs performance both within and beyond their training power ranges, providing a basis for a direct comparison of phase and power inference performance between different NNs trained on different datasets with varying power conditions, testing their ability to generalise to previously unseen power fluctuations.

To streamline the evaluation process, the upper boundary of the power range in the evaluation dataset was fixed at 1.00, whilst the lower boundary was incrementally raised from $\:{\rho\:}_{lower}=0.00$ in steps of 0.05 up to $\:{\rho\:}_{lower}=1.00$, resulting in 21 distinct datasets. In the dataset where the lower power boundary was $\:{\rho\:}_{lower}=0.00$, fibre power values varied uniformly at random within the range $\:\left[\text{0.00,1.00}\right]$; for the dataset with a lower boundary of $\:{\rho\:}_{lower}=0.05$, power values varied within the range $\:[0.05,\:1.00]$, and so forth, up to $\:\left[\text{1.00,1.00}\right]$, which represents no power variation (i.e., fixed power levels). Each dataset comprised 250 data pairs, each containing a Camera A observation of the intensity distribution and the corresponding phase and power values. The camera images were sequentially used as the input to the NNs. During NN inference, each dataset was processed individually, with all 250 examples evaluated to compute the mean phase and power prediction error for that dataset. These mean errors were then plotted against the corresponding lower boundary value of the power range. For instance, the mean error for the $\:\left[\text{0.00,1.00}\right]$ dataset was plotted at 0.00 on the x-axis, the mean error for the $\:\left[\text{0.05,1.00}\right]$ dataset at 0.05, and so forth. By processing every dataset incrementally, starting from $\:{\rho\:}_{lower}=0.05$ and ending with the $\:{\rho\:}_{lower}=1.00$, the evaluation spans all possible cases—from extreme power variations, including up to 100% fluctuations ($\:\left[\text{0.00,1.00}\right]$), to the ideal case of no power variations (i.e., $\:\left[\text{1.00,1.00}\right]$). This approach provides a comprehensive understanding of how different NNs, trained on varying power ranges, perform under different power fluctuation conditions.

The first NN considered in this work, referred to as NN^[1.0,1.0]^, was trained on a dataset with power levels fixed in the range $\:\left[\text{1.00,1.00}\right]$ (i.e., fixed at 100%). In contrast, the second NN, referred to as NN^[0.5,1.0]^, was trained on a dataset where power levels varied uniformly at random within the range $\:\left[\text{0.50,1.00}\right]$. Although this lower range limit may not agree with the typically observed power downgrade in HPFLs, it was chosen to evaluate the effect of including substantial power variations (up to 50%) in the training dataset on the ability of NN to generalise across different power distributions, particularly under conditions where output power fluctuates significantly.

**Fig. 4:**
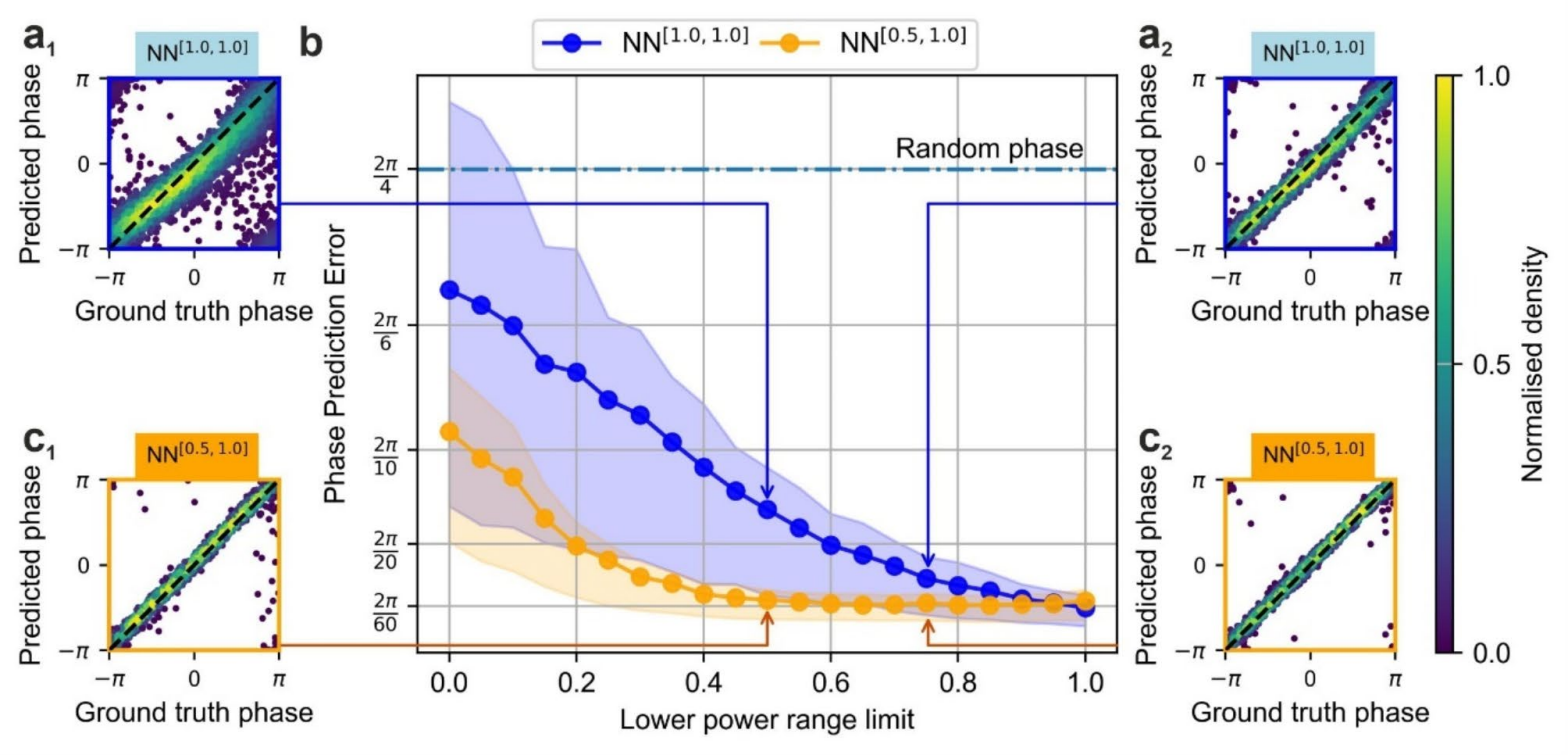
Prediction accuracies of the NNs evaluated on datasets with different power variation ranges. (**a**_**1**_) and (**a**_**2**_) show the prediction accuracies of NN^[1.0,1.0]^ when evaluated on datasets with power ranges [0.5,1.0] and [0.75,1.0], respectively. (**c**_**1**_) and (**c**_**2**_) show the prediction accuracies of NN^[0.5,1.0]^ when evaluated on datasets with power ranges [0.5,1.0] and [0.75,1.0], respectively. These evaluations correspond to the datapoints at 0.5 and 0.75 on plot (**b**), which illustrates phase prediction error as a function of the lower power range boundary $\:{\rho\:}_{lower}$ in the range [$\:{\rho\:}_{lower},1]$.

Consequently, NN^[1.0,1.0]^, trained on dataset with fixed power levels, encountered evaluation data pairs with varying power levels it had not previously seen (except for one evaluation dataset corresponding to the $\:\left[\text{1.00,1.00}\right]$ power variation range). In contrast, NN^[0.5,1.0]^, trained on dataset that included power variation in the range $\:\left[\text{0.50,1.00}\right]$, encountered evaluation datasets sharing more similarities to its training data, having not seen power ranges where the lower boundary was between 0.00 and 0.45. The results of these evaluations are presented in Fig. 4, with insets comparing ground truth phase versus predicted phase for the power ranges $\:\left[\text{0.50,1.00}\right]$ and $\:\left[\text{0.75,1.00}\right]$ (corresponding to the 0.50 and 0.75 data points on the x-axis, respectively). In each inset, the x-axis represents the ground truth phase applied to the SLM, whilst the y-axis shows the NNs predictions. Ideally, predicted phase values would match the ground truth values exactly, resulting in a $\:y=x$ line on a plot. Deviations from this line indicate discrepancies between the predicted phase values and the ground truth phase values, with a broader spread indicating reduced phase inference precision. The $\:y=x$ line is included in each inset for visual clarity.

Each phase inset (Fig. 4a_1–2_,c_1–2_) contains 1,500 data pairs from an evaluation dataset involving 250 intensity distributions captured from Camera A, combining phase inference results for the 6 outer beamlets. The insets for NN^[0.5,1.0]^ (Fig. 4c_1_,c_2_) show a consistent spread of phase values when tested on power ranges $\:\left[\text{0.50,1.00}\right]$ and $\:\left[\text{0.75,1.00}\right]$, with a mean phase prediction error of approximately $\:2\pi\:/60$, as shown in Fig. 3b. In contrast, NN^[1.0,1.0]^ exhibits a broader spread of predicted phase values (Fig. 4, a_1_, a_2_), with errors increasing from $\:4\pi\:/60$ at a lower boundary of 0.75 to approximately $\:8\pi\:/60$ at 0.50. This difference can be largely attributed to the ability of NN to interpolate and extrapolate unseen data. NN^[0.5,1.0]^, trained on a dataset with power variations spanning $\:\left[\text{0.50,1.00}\right]$, interpolates unseen, leading to more consistent and accurate phase predictions. In contrast, NN^[1.0,1.0]^, trained exclusively on fixed power levels $\:\left[\text{1.00,1.00}\right]$, encounters unseen data with lower power levels that fall outside its training range. This forces NN^[1.0,1.0]^ to perform extrapolation, predicting beyond the range of data it has been exposed to during training. Extrapolation is inherently more challenging than interpolation, often resulting in a broader spread of predicted values and higher phase prediction errors, as observed in Fig. 4(a_1_, a_2_). The increasing errors at lower power boundaries reflect the difficulty NN^[1.0,1.0]^ faces when generalising to unseen power variations, highlighting the limitations of training on fixed power levels. Whilst NN^[1.0,1.0]^ and NN^[0.5,1.0]^ perform similarly in the absence of power variations at maximum power as shown in Fig. 4b, the precision of NN^[1.0,1.0]^ degrades more quickly as power variation increases compared to that of NN^[0.5,1.0]^. Overall, NN^[0.5,1.0]^ predictions were more precise than NN^[1.0,1.0]^ across a broader range of power variations. This suggests that incorporating power variation into the training process enhances the robustness of the NN approach against power variations, rendering it more effective in realistic CBC systems where power degradation is often inevitable.

To further examine the impact of different power variation ranges included in the training on prediction accuracy, two additional NNs were trained on different power variation ranges. The first additional NN was trained on a dataset where power levels of all fibres varied uniformly at random within the range $\:\left[\text{0.80,1.00}\right]$ (hereafter referred to as NN^[0.8,1.0]^). This power fluctuation range is practically relevant, as a 20% power degradation is often observed over the lifetime of fibres due to various effects such as fibre photodarkening and/or pump failures. The second additional NN was trained on a dataset where power levels varied uniformly at random within the range $\:\left[\text{0.00,1.00}\right]$ (hereafter referred to as NN^[0.0,1.0]^). This broader power range represents an extreme case, simulating scenarios where at least one of channels experiences complete power failure. As a result, we have the previously introduced NN^[1.0,1.0]^ and NN^[0.5,1.0]^, along with the newly introduced NN^[0.8,1.0]^ and NN^[0.0,1.0]^. These two NNs NN^[0.8,1.0]^ and NN^[0.0,1.0]^, were tested on the same 21 datasets as NN^[1.0,1.0]^ and NN^[0.5,1.0]^.

**Fig. 5:**
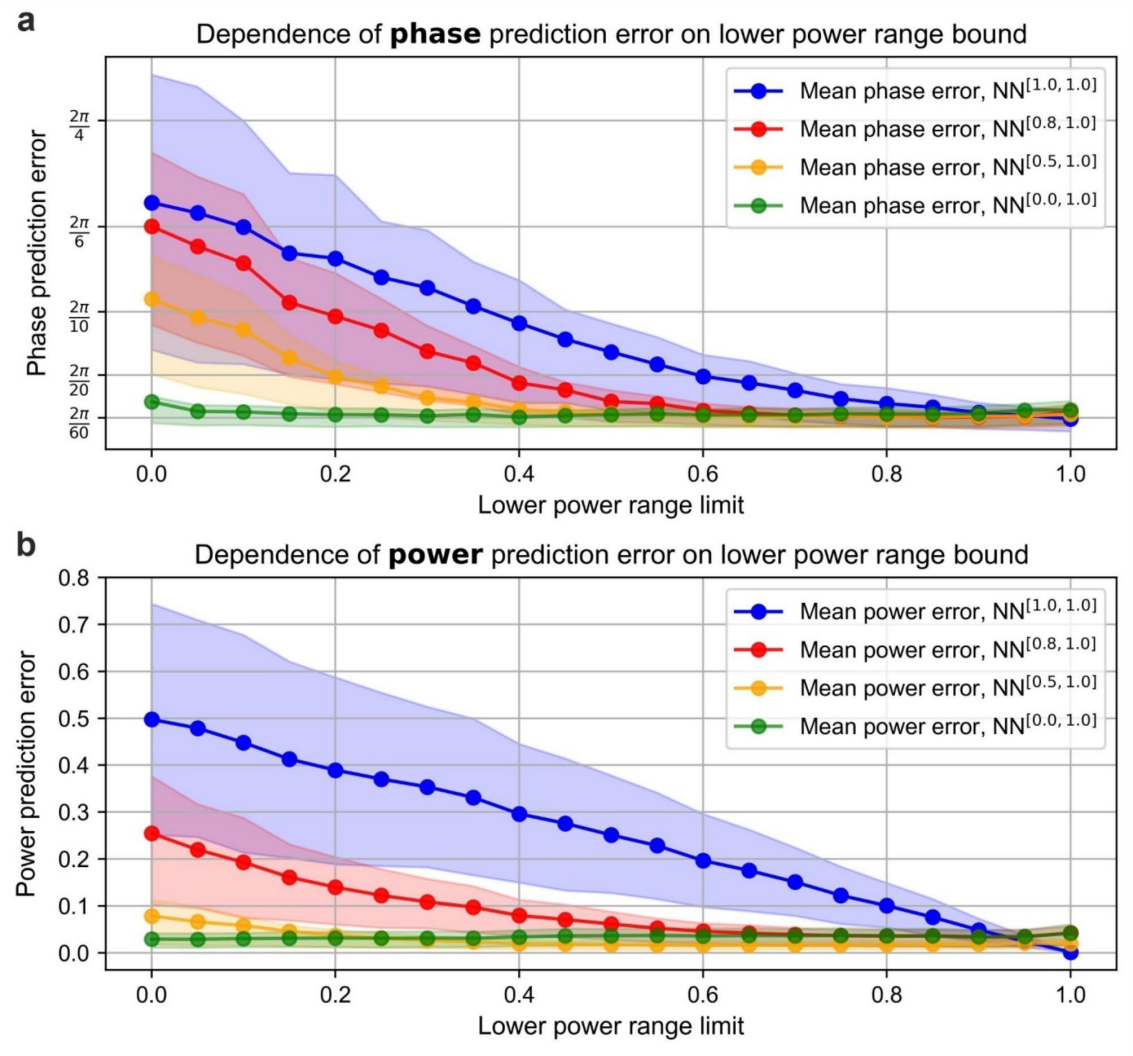
(**a**) Phase prediction error as a function of the lower power range limit $\:{\rho\:}_{lower}$, evaluated on datasets with varying power ranges using NNs trained on different power variation ranges. The lower boundary $\:{\rho\:}_{lower}$ was incrementally increased from 0.0 to 1.0 in steps of 0.05. (**b**) Power prediction error as a function of the lower power range limit $\:{\rho\:}_{lower}$, evaluated on the same datasets and under the same conditions as in (**a**).

Figure 5a shows the performance comparison of NN^[1.0,1.0]^, NN^[0.5,1.0]^, NN^[0.8,1.0]^, and NN^[0.0,1.0]^. In the $\:[1.00,\:1.00]$ power range (i.e., power fixed at 100% with no power variation), mean phase prediction errors were 0.10, 0.12, 0.13, and 0.14 radians for NN^[1.0,1.0]^, NN^[0.5,1.0]^, NN^[0.8,1.0]^, and NN^[0.0,1.0]^, respectively. Evidently, NN^[1.0,1.0]^ performed best in this range (i.e., $\:\left[\text{1.00,1.00}\right]$), which could be explained by the fact that its training dataset comprises solely of intensity distributions collected within this power level range. Conversely, NNs trained on varying power ranges ($\:\left[\text{0.00,1.00}\right]$, $\:\left[\text{0.50,1.00}\right]$, $\:\left[\text{0.80,1.00}\right]$) exhibited slightly higher inference errors in the $\:\left[\text{1.00,1.00}\right]$ test range. This outcome can be attributed to the fact that their training involved random and uniform variations in power levels, making it unlikely to encounter data points where all power levels are equal. As a result, these NNs were less exposed to high fringe visibility during training, which reduced their inference precision when evaluated in the fixed power range $\:\left[\text{1.00,1.00}\right]$. In the power range with the lowest lower bound (i.e., $\:\left[\text{0.00,1.00}\right]$), NN^[0.0,1.0]^ demonstrated the highest precision, followed by NN^[0.5,1.0]^, NN^[0.8,1.0]^, and NN^[1.0,1.0]^, with phase prediction errors of 0.18, 0.69, 1.04, and 1.17 radians, respectively. This indicates that NNs trained with broader power variations are more robust under fluctuating power conditions.

Interestingly, although all four NNs discussed here were trained on specific power variation ranges, their performance does not degrade abruptly beyond these ranges. Specifically, NN^[1.0,1.0]^, trained on a dataset with no power variations, shows a similar phase prediction error when tested on the $\:\left[\text{1.00,1.00}\right]$ power range as when tested on the $\:\left[\text{0.90,1.00}\right]$ range, after which it begins to degrade rapidly. This suggests that NN^[1.0,1.0]^ can maintain accurate phase inference even with a previously unencountered 10% power variation. Likewise, NN^[0.8,1.0]^ and NN^[0.5,1.0]^, trained on the $\:\left[\text{0.80,1.00}\right]$ and $\:\left[\text{0.50,1.00}\right]$ power ranges, maintain phase prediction accuracy up to $\:\left[\text{0.70,1.00}\right]$ and $\:\left[\text{0.40,1.00}\right]$, respectively, before their accuracy begins to decline, indicating a similar tolerance of approximately 10% beyond their trained range.

This pattern suggests that all NNs share a similar ability to extrapolate beyond their respective training ranges, with a tolerance of approximately 10% extension. However, the NNs trained on datasets with greater power variations ($\:\left[\text{0.50,1.00}\right]$ and $\:\left[\text{0.80,1.00}\right]$) demonstrated better overall performance under more challenging power fluctuation conditions. This observation indicates that incorporating broader power variations during training increases robustness to amplitude noise and enables better generalisation. By exposing the NN to a wider range of power distributions during training, the NN learns to handle the variability inherent in real-world CBC systems more effectively. The exception to the previously considered NNs is NN^[0.0,1.0]^, trained on the full power variation range. Unlike the other NNs, it only interpolates, as it has already seen the entire range during training. A slight decrease in precision is observed when tested on the $\:\left[\text{0.00,1.00}\right]$ power fluctuation range. This is due to some fibres being effectively turned off, making phase prediction for those fibres akin to a random guess.

The same analysis was conducted for power predictions of the NNs, using the same set of evaluation datasets as in the phase analysis, with the results shown in Fig. 5b. Whilst the general behaviour of the curve remained similar to the phase case, several distinct differences were observed. Firstly, the power prediction error of the NN^[1.0,1.0]^ remains constant. This is because, although power information was included in the training dataset, it was fixed at the level of 1.00. As a result, the NN effectively learnt to predict a constant value of 1.00, with no gradient in the training data to correlate with varying power levels. Therefore, when tested on datasets with varying lower power limit, the power prediction error follows $\:(1-{\rho\:}_{lower})/2\:$, decreasing linearly as $\:{\rho\:}_{lower}$ approaches 1.00, with the error reaching 0 when $\:{\rho\:}_{lower}=1.00$. On the other hand, NN^[0.8,1.0]^ and NN^[0.5,1.0]^ demonstrated a greater ability to generalise power predictions (compared to their generalisation of phase), as evidenced by a slower decrease in accuracy and precision as the lower power limit decreases. In particular, when tested on the dataset with a power variation range of $\:\left[\text{0.00,1.00}\right]$, the mean power prediction error was 0.25 and 0.08 for NN^[0.8,1.0]^ and NN^[0.5,1.0]^, respectively. Finally, NN^[0.0,1.0]^ demonstrated a nearly constant power prediction error across all power variation ranges, starting at 0.04 when tested on $\:\left[\text{1.00,1.00}\right]$ and decreasing slightly to 0.03 when tested on $\:\left[\text{0.00,1.00}\right]$. This lack of increase in power prediction error, even under extreme power variations (i.e., with a lower power range limit of 0.00), contrasts with the phase prediction case for NN^[0.0, 1.0]^. This can be attributed to the fact that, for power, unlike phase, the absence of a signal still carries explicit information about the system state since it corresponds to a measurable intensity value. In contrast, phase cannot be determined in the absence of a signal, as it relies on the interference between multiple beamlets to extract information. Although the power of each beamlet can be measured precisely by tapping out a small amount and monitoring it individually with a photodiode detector, the analysis above demonstrates that this additional power monitoring can be effectively replaced by the NN, thereby simplifying the experimental setup and reducing hardware requirements.

Overall, these findings highlight the trade-off between training range and accuracy in phase and power inference. Whilst narrower power variation ranges yield slightly higher accuracy within their respective ranges, the improvement is not significantly greater and remains somewhat comparable to NNs trained on broader ranges. On the other hand, incorporating broader power variations improves robustness to amplitude noise and enhances the ability of NN to generalise across real-world power fluctuation scenarios. This demonstrates that a well-trained NN can accurately interpret subtle variations in intensity interference patterns and correctly associate them with either phase or amplitude changes. This balance is particularly critical for practical CBC systems, where unpredictable amplitude noise and power degradation cannot be dismissed over time.

### Scalability of the CNN approach

Previous results demonstrate that the NN approach is able to accurately and precisely identify phases and powers in a single-step for a 7-beam, hexagonally closed-packed beamlet array from a single camera observation of its intensity distribution. However, investigating the performance and applicability of the NN approach as the number of beamlets in a CBC array increases remains an area of significant interest.

As the number of beamlets in a CBC system increases, the number of phase interactions between adjacent and non-adjacent beamlets grows, thereby amplifying the complexity of the resulting interference patterns. Consequently, the NN might need to process increasingly intricate interference patterns to accurately and precisely extract phase and amplitude information, which could necessitate larger datasets to maintain comparable performance. For a CBC system with N beamlets, there are $\:N\cdot\:(N-1)/2$ unique interactions between beamlets, suggesting that the number of training pairs could potentially increase at a rate comparable to the number of these interactions. This raises the possibility that the size of the required dataset could increase quadratically with the number of beamlets. From a practical perspective, quadratic scaling is undesirable, as it would render the approach less feasible for CBC systems with a larger number of composing beamlets. This raises an important question about whether the NN approach is applicable to a CBC array with a higher number of beamlets without compromising its ability to extract phases and powers efficiently. Therefore, it is essential to determine whether the dataset requirements scale up linearly, quadratically, or at an intermediate rate as the number of beamlets increases. To address this question, we first examine the phase prediction error achievable for a given number of training pairs for a given number of beamlets in CBC system. Then, we examine the number of training pairs required for a given CBC array to achieve a targeted level of phase prediction accuracy. This process is then repeated for power prediction error. Together, these analyses provide insight into the scalability of the NN approach and determine whether it remains practical for CBC problems as the number of beamlets increases.

**Fig. 6:**
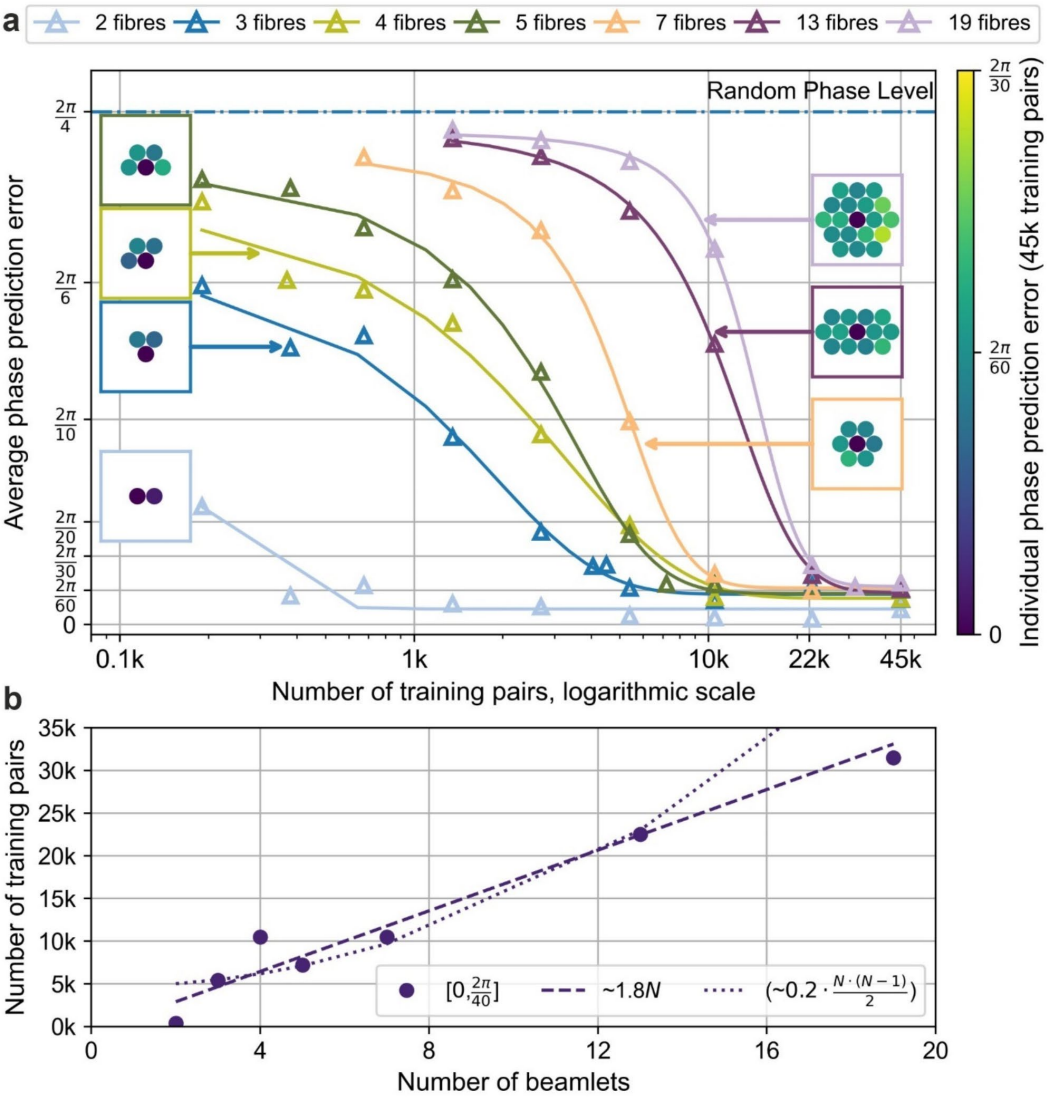
(**a**) Curves illustrating the average phase prediction error after the final epoch of NN^[0.5,1.0]^ training, plotted as a function of the number of training pairs for different CBC arrays. Each curve corresponds to a specific number of fibres, (**b**) Minimum number of training pairs required to achieve phase precision within the range $\:[0,\frac{\pi\:}{20}]$ as a function of the number of fibres. The dashed line represents a linear fit, whilst the dotted line corresponds to the $\:{k}_{2}\cdot\:N\cdot\:(N-1)/2$ fit.

To gain insight into the scalability of the NN approach for fibre arrays with a greater number of beamlets, training data were collected for fibre arrays consisting of 2, 3, 4, 5, 13, and 19 beamlets, in addition to the previously collected datasets for the 7-fibre system. The fibre arrays, shown as insets in Fig. 6a, were derived from the original hexagonal close-packed array initially used for the 7-fibre system, with beamlets omitted or added as needed to achieve the required number of composing beamlets. The radii of all the beamlets remained the same as those in the previously considered 7-fibre system, ensuring consistent physical parameters across all CBC arrays. During training data collection for all CBC arrays, the phase of all beamlets (except for the designated reference beamlet) varied in the range $\:[-\pi\:,\pi\:]$, while the power levels of all beamlets were set in the range $\:\left[\text{0.5,1.0}\right]$. These ranges are consistent with the dataset used for NN^[0.5,1.0]^ training in the earlier analysis of the 7-fibre system. The lower power limit of 0.5 was chosen here as a balance point between 0% and 100% power. The idealised dataset $\:\left[\text{1.0,1.0}\right]$ used for the training of NN^[1.0,1.0]^, required NN^[1.0,1.0]^ to extrapolate across the entire range of power variations, whereas the dataset with extreme power variations (i.e., ranging from 0 to 1) used for NN^[0.0,1.0]^ required it to perform interpolation only. Compared to the lower power limit of 0.8, 0.5 was preferred as a midpoint between these two extremes, offering a practical compromise that combines generalisability and robustness.

To assess the phase prediction error achievable with a given number of training pairs, a dataset of 50,000 pairs was collected for each CBC array. Consistent with the previous methodology, the dataset for each CBC array was split into 90% for training and 10% for validation, yielding 45,000 training pairs for NN training and 5,000 pairs for NN validation. The NN for each beamlet array was first trained using 45,000 training pairs. Its performance was evaluated after each epoch using the 5,000-pair validation set, and the mean prediction error from the final epoch was recorded as a measure of performance for the dataset. Subsequently, the size of the training dataset, starting with 45,000 pairs, was iteratively reduced by approximately half over multiple iterations by randomly selecting data pairs to retain. In the first iteration, it was reduced to 22,500 pairs. A new NN with randomly initialised weights was trained on this reduced dataset and evaluated using the same 5,000-pair validation set as before. After each epoch, the NN was validated on this validation set, and the mean prediction error was recorded after the final epoch. This halving process continued, progressively reducing the training set, creating a new data point at each iteration to illustrate the relationship between phase prediction error and the number of training pairs for a given CBC array.

As a result of this iterative reduction in dataset size, curves illustrating the phase prediction error as a function of the number of training pairs were obtained for CBC arrays with different numbers of beamlets. These curves exhibit a characteristic S-shaped trend, as shown in Fig. 6a, reflecting the performance of all NNs trained for CBC arrays, measured in terms of phase prediction error. When datasets with heavily reduced sizes were provided to the NNs as training materials, the prediction error remained relatively high, indicating an insufficient number of data for the NN to acquire a generalised ability to extract phase information from interference patterns between beamlets. As the number of training pairs increased, the error decreased significantly, marking a rapid improvement in the performance of the NNs during the learning stage. This stage corresponds to the NNs acquiring the ability to infer phase information that generalises well to unseen data. However, beyond a certain number of training pairs (e.g., 11,000 for the 7-fibre CBC array), the improvement in performance diminished, and the curves reached a plateau. This plateau suggests that the NN had effectively saturated its learning capacity for the given data, achieving near-optimal performance with limited additional improvement from further increasing the size of the training dataset. The observed behaviour is consistent with the typical S-shaped curves in machine learning, where mean prediction error improves as a function of number of training pairs. These curves characterised by an initial stage where data scarcity limits performance, followed by a growth stage and eventual saturation as the NN approaches its learning capacity.

Figure 6a includes insets showing the individual average phase prediction errors for each beamlet in CBC arrays with 2, 3, 4, 5, 7, 13, and 19 fibres, when each corresponding NN was trained with 45k training pairs. These insets map beamlet positions and error magnitudes, revealing no strong positional bias (e.g., outer vs. central beamlets). This uniformity highlights the ability of the NN to generalise phase extraction across the array, despite increasing pattern complexity.

From Fig. 6a, it is evident that as the number of beamlets increases, the plateau in the S-shaped curve occurs at progressively higher numbers of training pairs. This indicates that, for CBC arrays with a higher number of beamlets, a greater number of training pairs is required for the NN to reach saturation in phase prediction accuracy and achieve near-optimal performance. This observation aligns with the previously discussion: introducing more beamlets increases the number of interference interactions between them, resulting in more intricate patterns that necessitate larger datasets for the NN to extract phases and powers with comparable precision and accuracy. The remaining question is to determine the rate at which the requisite number of training pairs for enabling a trained NN with satisfactory level of accuracy and precision in extraction phase and amplitude information grows as the number of beamlets increases.

To determine the rate at which the number of training pairs grows with the number of fibres, we analysed the minimum number of training pairs required to achieve a specific phase prediction error for each CBC array. A horizontal slice was taken across Fig. 6a at a phase prediction error range of $\:[0.0,\:\pi\:/20]$. This range was chosen because it ensures a good level of precision that the NN should aim to achieve. When the phases of all beamlets (except the central one) vary with a zero mean and a standard deviation of $\:\pi\:/20$, approximately 99.0% of the expected power remains in the bucket within the central lobe for the considered CBC arrays. For each CBC array, the minimum number of training pairs required to reach this precision was identified and plotted against the corresponding number of fibres, as shown in Fig. 6b. The data points were then fitted with two functions: $\:{F}_{1}\left(N\right)={k}_{1}N+{c}_{1}$ and $\:{F}_{2}\left(N\right)={k}_{2}\cdot\:N\cdot\:(N-1)/2+{c}_{2}$, where $\:N$ is the number of beamlets, $\:{k}_{1}$, $\:{k}_{2}$, and $\:{c}_{1}$, $\:{c}_{2}$ are fitting coefficients. The former function, $\:{F}_{1}\left(N\right)$, represents a linear fit, with the first 6 data points used to determine the optimal fitting coefficients $\:{k}_{1}$ and $\:{c}_{1}$. Similarly, for the latter function, $\:{F}_{2}\left(N\right)$, coefficients $\:{k}_{2}$ and $\:{c}_{2}$ were also determined using the first 6 data points. The fitted curve was then compared against the last data point to assess whether it lies below or above the fit, providing insight into whether the required number of data points, for the NN approach, grows linearly or quadratically for a higher number of beamlets in a CBC system. Figure 6b presents both the linear fits, as shown in the dashed line and the quadratically fits as shown in dotted line. The linear fits evidently more closely resemble the trajectory of the datapoints. Thus, indicating that a linear scaling of the required training pairs may suffice for achieving the desired phase precision. These results demonstrate that the NN approach could be applied to CBC systems with higher number of beamlets, without necessitating the collection of a prohibitive number of data pairs.

**Fig. 7:**
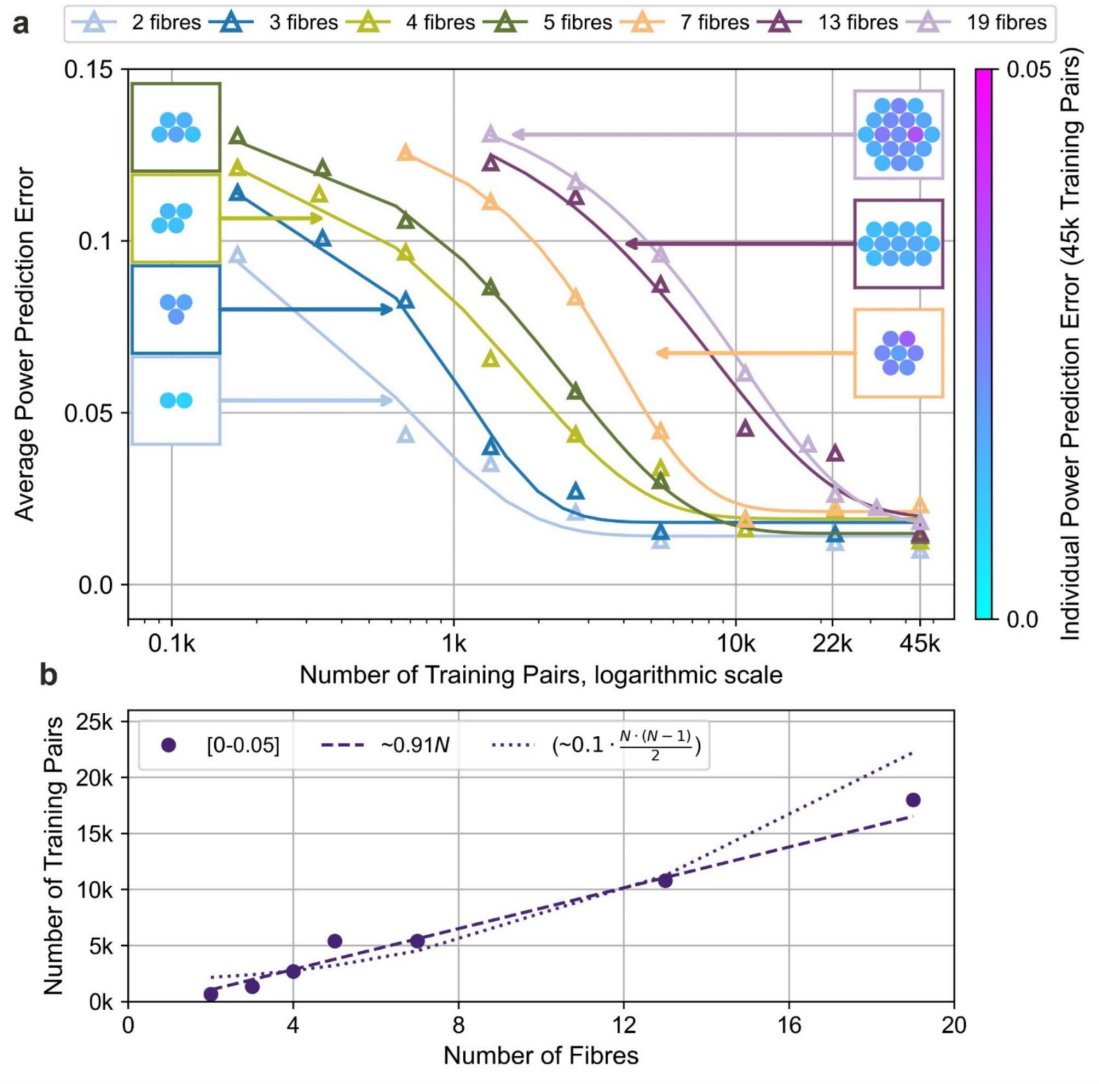
(a) Curves showing the average power prediction error after the final epoch of NN^[0.5,1.0]^ training, plotted against the number of training pairs for different CBC arrays. Each curve represents a specific number of fibres., (b) Minimum number of training pairs needed to achieve power precision within the range [0, 0.05], shown as a function of the number of fibres. The dashed line indicates a linear fit to the data, while the dotted line corresponds to the $\:{k}_{2}\cdot\:N\cdot\:(N-1)/2$ fit.

The analysis above was repeated for power predictions, as shown in Fig. 7a, b. Figure 7a includes insets displaying individual average power prediction errors per beamlet for CBC arrays with 2, 3, 4, 5, 7, 13, and 19 beamlets, trained with 45k pairs each. These insets map beamlet positions and errors, showing no clear correlation with position (e.g., outer vs. central beamlets); intriguingly, outer beamlets generally exhibit lower errors, an observation warranting further investigation. Similar to the phase analysis, the results for power indicate that the data points align more closely with the linear fit, suggesting that the required number of training pairs for accurate power predictions also scales linearly with the number of fibres. However, the number of training pairs required to achieve precision within a 5% error (0.05 for power and $\:2\pi\:/40$ for phase, equivalent to a 5% error) scales with a lower gradient for power than for phase.

We hypothesise that this near-linear scalability arises from recording intensity distributions in a plane away from the focal plane, where each beamlet partially overlaps primarily with its immediate neighbours. Such localised overlap reduces global interference complexity and allows NN to isolate each beamlet’s contribution (see Supplementary Material 5).

We acknowledge that the results presented in this manuscript are based on a simulated setup using a spatial light modulator, which approximates the ideal intensity distributions of a tiled-aperture CBC fibre system. However, real-world CBC systems are subject to additional error sources, such as thermal drift, mechanical vibrations, and installation errors (e.g., tip-tilt misalignments), which are not explicitly accounted for in our current setup. These factors may impact the practical adoption of our method. Nevertheless, we view our findings as an important first step toward realising a more effective fibre-based CBC implementations, which we aim to explore in future research.

## Conclusions

In this work, we have demonstrated the capability of the NN approach to simultaneously infer both phase and power information from a single camera observation of the intensity distribution in a single step. By accurately interpreting subtle variations in intensity interference patterns, the NN can distinguish between phase and power contributions without requiring separate measurement systems (e.g., photodiode power sensors). This streamlined and efficient approach not only simplifies the measurement process but also enhances the precision of phase and power monitoring, making it highly suitable for practical applications in CBC systems.

We further investigated the robustness of phase inference under power degradation, a common phenomenon over the operational lifetime of fibre lasers. Our analysis highlighted the potential impact of inevitable power variations on phase prediction accuracy, providing insights into the long-term reliability of the NN-based system.

Additionally, we explored the scalability of the NN approach by analysing its performance across CBC systems with varying numbers of beamlets. We showed that the NN requires a number of training pairs that scales linearly with the number of beamlets to achieve a predetermined level of phase and power prediction accuracy. This linear scaling suggests the practicality of the NN approach for larger CBC systems.

These findings collectively underscore the potential of the NN approach as a scalable, accurate, and efficient solution for phase and power inference in CBC systems, paving the way for its integration into high-power fibre laser systems with increased beamlet counts.

## Electronic supplementary material

Below is the link to the electronic supplementary material.

Supplementary Material 1

## Data Availability

The datasets generated and/or analysed during the current study are available in the DATASET repository, https://doi.org/10.5258/SOTON/D3389.
