# Supplementary material for: Deep learning for simultaneous phase and amplitude identification in coherent beam combination
Source: Sci Rep. 2025 Apr 6;15:11757. doi: 10.1038/s41598-025-96385-w (PMC11973209; doi:10.1038/s41598-025-96385-w)
Supplement: Supplementary file 1 — Supplementary Material 1 [file 41598_2025_96385_MOESM1_ESM.docx]

**Supplementary Information**

Fedor Chernikov*^1^, Yunhui Xie^1^, James A. Grant-Jacob^1^, Yuchen Liu^1^, Michalis N. Zervas^1^, Ben Mills^1^
^1^ Optoelectronics Research Centre, University of Southampton, UK.
* fc1e21@soton.ac.uk

## Real Time Phase Correction

In the main manuscript, Figure 6a provides insights into the scalability of the NN approach, leading to its continuation in Figure 6b. However, another significant observation can be drawn from Figure 6a. Whilst Figure 6a,b examine the scalability, the analysis here focuses on the phase prediction error at the point where all S-curves have converged—specifically, when trained with 45,000 pairs, representing near-optimal performance of the NN. An additional key insight, highlighted in Supplementary Figure 1a, is the increase in phase prediction error as the number of beamlets increases. This observation is further detailed in Supplementary Figure 1b, demonstrating that phase prediction accuracy decreases logarithmically with an increasing number of fibres. For instance, the prediction error is 0.05 radians for a 2-fibre system but increases to 0.13 radians for a 19-fibre system. Whilst this decline in accuracy is gradual, it raises potential concerns about the scalability of the approach. Specifically, as the system size grows and more fibres are added, the reduced prediction accuracy may limit the effectiveness of the method in larger fibre arrays.


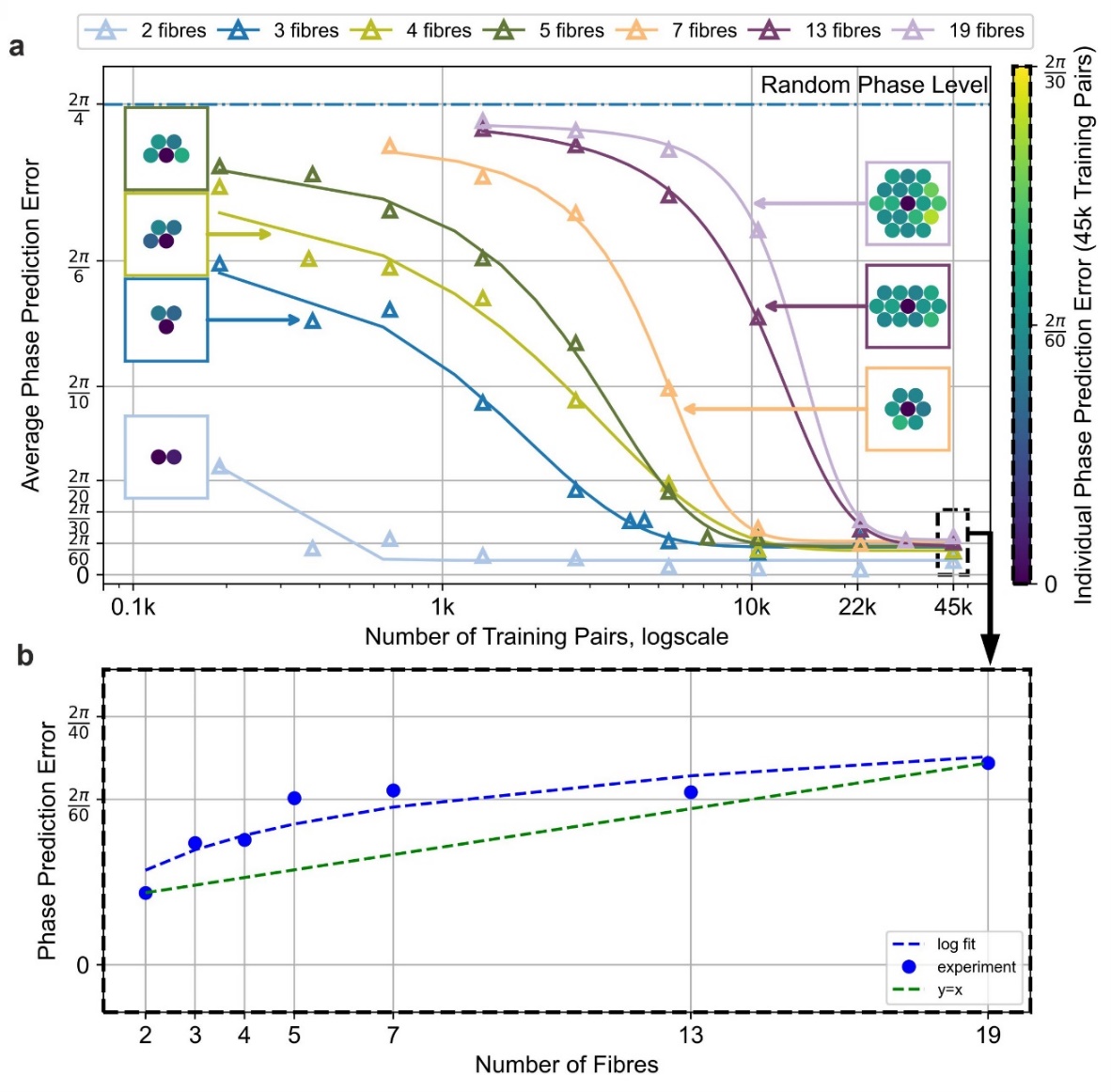


Supplementary Figure 1. Phase prediction error as a function of the number of beamlets, obtained from testing a neural network trained on 45,000 training pairs. The number of training pairs was chosen at the point where all S-curves reached their plateau, ensuring optimal network performance.

To evaluate the impact of increasing phase prediction error in CBC systems with higher count of beamlets, real-time correction was performed for the 3-, 7-, and 19- fibre configurations. These configurations were selected due to their symmetry, which, when all fibres are in phase (also referred to as ‘flat-phase’) produces a symmetrical beam profile with the brightest spot in the centre and side lobes around it. Such symmetrical beam profiles allow for the use of a widely accepted performance metric, Power in the Bucket (PIB), to evaluate the effectiveness of the NN. For the real-time correction, NN models trained specifically for the 3-, 7-, and 19-fibre configurations introduced in the ‘Scalability of the CNN Approach’ subchapter were implemented.

Supplementary Figure 2a_1_ demonstrates how regions for calculating PIB were defined taking as an example 7-fibre CBC array. For each fibre configuration (i.e., 3, 7, and 19 beamlets), flat-phase, equal-power values (set to 1) were applied to the SLM. The resulting ‘flat-phase’ far-field intensity pattern, labelled as ‘Reference’ in the Supplementary Figure 2a_1_ was recorded on Camera B. The “bucket” radius was determined by measuring the distance from the pixel with maximum intensity in the central spot to the pixels with half of that maximum intensity (i.e., half of the Full Width Half Maximum, or FWHM, as indicated in Supplementary Figure 2a_1_). This radius defined the circular "bucket" region, centred on the pixel with maximum intensity, where power was monitored. The bucket size was individually determined for each of the three CBC configurations, as the central spot size decreased with increasing count of beamlets. The PIB of the reference flat-phase intensity pattern was defined as 100%, and the normalised PIB of the beams corrected during real-time control was calculated relative to this reference value. The Supplementary Figure 2a_2_ shows two observations from Camera B, both highlighting the "bucket" region with a black circle, where power was calculated by summing all pixel values within the bucket region. The first image labelled as “Reference” represents "flat-phase" intensity distribution, with the circle illustrating the relative position of the intensity distribution and the "bucket" region. The second image labelled as “Timestep N” shows the intensity distribution at timestep N collected during real-time correction, corresponding to an unknown set of phases. Unlike the reference camera observation, the intensity distribution from the second camera observation shows noticeable variation, illustrating the deviation caused by the unknown set of phase values. Together, these camera observations help visualise the comparison between the reference and the possible distribution at Timestep N.

The real-time correction procedure was similar to the one used in [1] with schematic illustrated in the Supplementary Figure 2b and goes as follows: At timestep = 0, random phase values (referred to as "True Phase" in Supplementary Figure 2b) were applied to the SLM panel, with the power levels of all fibres fixed at 1 for simplicity. The corresponding intensity distribution was recorded by Cameras A and B. The image from Camera A was provided as input to the pretrained NN, which identified the fibre phases (referred to as "Predicted Phase" in Supplementary Figure 2b). The Camera B observation, despite being shown in Supplementary Figure 2b, was used solely to observe the far-field pattern and was not input into NN. The predicted phases were then subtracted from the initial phase values, and random phase noise $\mathcal{N}$ was introduced. The noise was randomly selected from a normal distribution $\mathcal{N}\left( \mu,\sigma^{2} \right)$, with the standard deviation $\sigma$ incrementally increased, starting from 0π (no phase noise) and gradually rising up to $2\pi$ in steps of $\pi/20$. For simplicity in comparing different standard deviations, a mean of zero was chosen. Supplementary Figure 2 illustrates an example of real-time correction when $\mathcal{N}\left( \mu=0,\sigma^{2}=5\pi/20 \right)$. After the new set of phase values was calculated, it was sent to the SLM panel, the resulting pattern was recorded by Camera A, and later provided as an input to the NN. The NN then identified the new set of phase values, and another round of phase noise was applied. This process was repeated for 9 steps, after which a new random initial phase pattern was displayed, and the entire procedure was repeated.

To further evaluate the performance of the NNs, an additional test was conducted with schematic shown in Supplementary Figure 2c. In this test, a flat-phase was set on the SLM at timestep = 0, producing an ideal "flat-phase" far-field intensity pattern. Random noise, sampled from a normal distribution with zero mean and a standard deviation incrementally increasing from $0$ to $2\pi$ in steps of $\pi/20$, was then added to the flat-phase. By introducing random noise to the flat-phase at each timestep, an ideal NN prediction was simulated. Essentially, whilst the NN control was applied (Supplementary Figure 2b), the following phase manipulation occurred: (True phase state – Phase prediction + Phase noise). However, in the scenario depicted in Supplementary Figure 2c, this is simplified to (Flat-phase + Phase noise), where the Flat-phase equals the difference between True phase and the Phase prediction—applicable only in cases of perfect phase prediction by the NN.


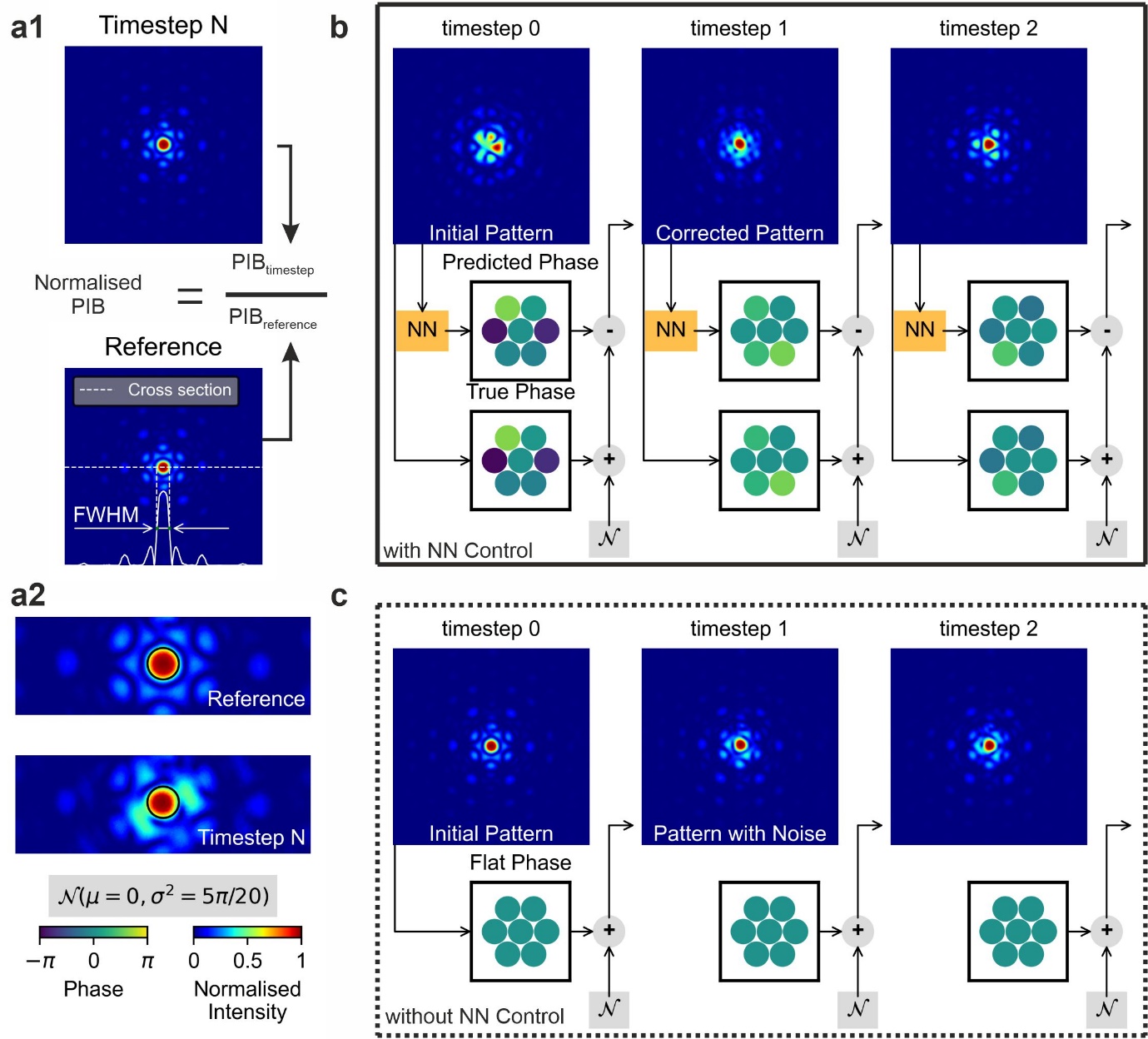


Supplementary Figure 2. a) An example of how Power In the Bucket was defined, b) Schematic of real-time control with Neural Network implemented to make phase predictions based on the current timestep combined intensity distribution, c) Schematic of real-time control without Neural Network, at every timestep recorded intensity distribution corresponds to a phase profile which is a sum of ‘flat-phase’ and simulated noise, effectively replacing an ideal NN prediction.

Supplementary Figure 3(a, b, c) illustrates how PIB degrades as phase noise is incrementally increased between each step when the camera observation is captured for 3, 7 and 19 CBC configurations. A key insight from this graph is that, despite the decreasing phase prediction accuracy observed in Supplementary Figure 1c, the degradation does not significantly impact practical application performance. All curves begin near a normalized PIB value of 1, indicating that initial correction performance remains robust across all configurations. Specifically, the PIB starts at 99.8% (Supplementary Figure 3a) for the 3-fibre configuration (indicating effective correction from a random pattern to a 'flat-phase' pattern without phase noise), 99.5% (Supplementary Figure 3b) for the 7-fibre configuration, and 98.4% (Supplementary Figure 3c) for the 19-fibre configuration. At the highest noise level, where the noise has a greater influence on the pattern than the corrections applied by the CNN, the PIB levels drop to approximately 84% for 3 fibres, 55% for 7 fibres, and 28% for 19 fibres. All curves obtained with NN control (following the sequence shown in Supplementary Figure 2b) closely follow those obtained without (following the sequence shown in Supplementary Figure 2c, representing ideal NN prediction), confirming the near-perfect efficiency of the CNN in phase correction. Given the observed logarithmic trend in the decrease of phase prediction accuracy for configurations with a higher number of fibres, similar correction precision approaching 100% can be expected with a fine-tuned network. This suggests that the method employed here is efficient, even for more complex systems with a greater number of fibres.

Another notable observation from Supplementary Figure 3 is that the PIB for the 19-fibre system declines more quickly than for the 7- or 3-fibre systems. This suggests that, in a system experiencing real phase noise, maintaining a certain PIB level requires lower prediction latency for the 19-fibre configuration compared to the 7-fibre one, and similarly lower latency for 7 fibres compared to 3 fibres. Assuming the phase noise accumulates at the same rate regardless of the number of fibres—which is reasonable, given that each channel is individually amplified—the system can tolerate a higher phase noise level for 7 fibres than for 19 whilst still maintaining a desired PIB percentage. Based on experimental results, to maintain a PIB of 97% or above with the given networks for a given configuration, the allowed phase noise levels are $4\pi/20$ for the 3-fibre configuration, $3\pi/20$ for the 7-fibre configuration, and $\pi/20$ for the 19-fibre configuration. This implies that the prediction time can be longer for the 7-fibre configuration than for the 19-fibre configuration, and longer for 3-fibre configuration than for 7-fibre configuration. However, this contrasts with typical practice, where more computationally intensive networks are required for accurate phase prediction in systems with a larger number of fibres. For the 19-fibre system, predictions were achieved with a 1082 Hz bandwidth, compared to 1500 Hz for the 7- and 3-fibre systems. Performance measurements were recorded on a Microsoft Windows 10 machine with an Intel Xeon W5-3423 CPU and an NVIDIA RTX A4000 GPU, utilising the Open Neural Network Exchange (ONNX) format with the TensorRT Execution Provider to accelerate the model. The inference time accounted solely for NN predictions, excluding file reading and loading times, which further slowdown the process. Including these additional steps, the effective inference bandwidth drops significantly to 571 Hz for the 19-fibre system and 831 Hz for the 7- and 3-fibre systems, respectively. At present, latency appears to be the primary limitation for the effective implementation of CNNs in this context.


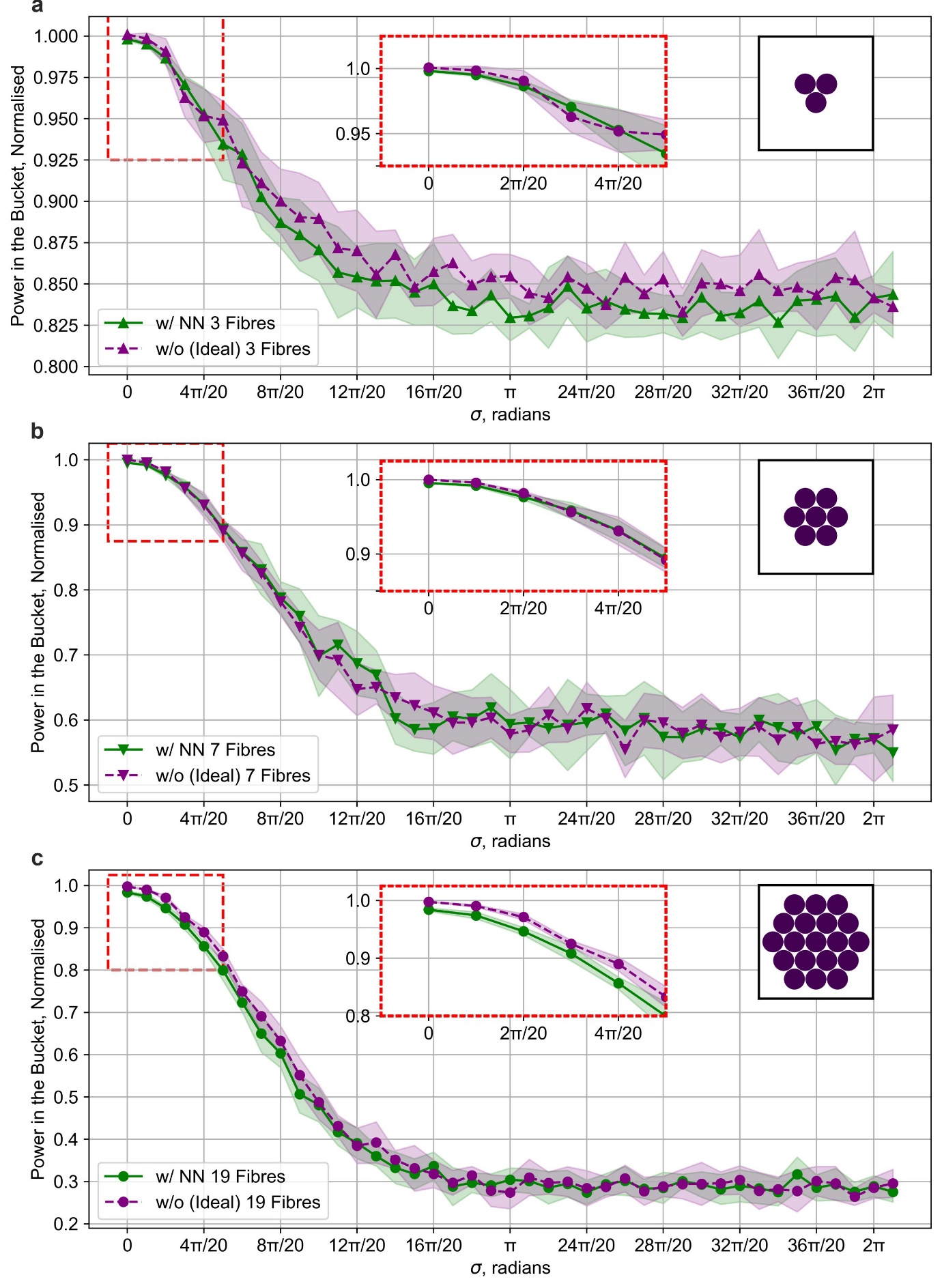


Supplementary Figure 3. Power in the Bucket as a function of the standard deviation for (a) 3 fibres, (b) 7 fibres, and (c) 19 fibres. The green line illustrates the performance of the NN in phase correction, while the purple line represents the ideal performance theoretically achievable by the NN.

## Neural Network Specifications

Table 1 Training parameters for Neural Networks used for phase and amplitude inference from a single camera observation of intensity distribution generated by a CBC array with different number of beamlets.

|  | 2 fibres | 3 fibres | 4 fibres | 5 fibres | 7 fibres | 13 fibres | 19 fibres |
| --- | --- | --- | --- | --- | --- | --- | --- |
| CPU | Intel i7-7700 | Intel i7-6700 | Intel i7-7700 | Intel i7-6700 | Intel i7-7700 | | Intel i7-6700 |
| GPU | Nvidia Quadro P6000 | Nvidia Titan X | Nvidia Quadro P6000 | Nvidia Titan X | Nvidia Quadro P6000 | | Nvidia Titan X |
| epochs | 200 | | | | | | 300 |
| Number of training pairs | 50,000 | | | | | | |
| Training time | 20 hours 30 minutes | 13 hours 48 minutes | 21 hours 27 minutes | 15 hours 23 minutes | 13 hours 21 minutes | 17 hours 45 minutes | 10 hours 37 minutes |
| Network | MobilenetV3 Small | | | | | MobilenetV3 Large | |

## Relative performance of model architectures

MobileNetV3-Small was chosen over other popular architectures, such as ResNet and Vision Transformer (ViT)[2], due to its improved computational efficiency. MobileNetV3 is specifically designed for low latency and reduced computational requirements. Comparatively, ResNet relies on standard convolutions, which are computationally more expensive, whilst ViT employs self-attention mechanisms that could lead to a higher computational demand due to quadratic scaling with number of tokens. MobileNetV3 addresses the latency challenges by utilising depthwise separable convolutions, which drastically reduce the number of parameters and floating-point operations without compromising performance.

To validate this architectural choice, we conducted a comparative study by training ResNet‑18 and ViT-base on the same dataset (corresponding to the 7-beamlet CBC system) used for MobileNetV3 (referred to as NN^[0.5,1.0]^ in the main manuscript). The training procedure was kept consistent across all models to ensure a fair comparison. Specifically, a dataset comprising 50,000 training pairs was split into training and validation sets with a 90/10 ratio. Furthermore, to maintain consistency with prior evaluations, we employed datasets with gradually increasing lower power limits, $\rho_{lower}$, enabling a systematic assessment of the models' robustness under varying conditions.


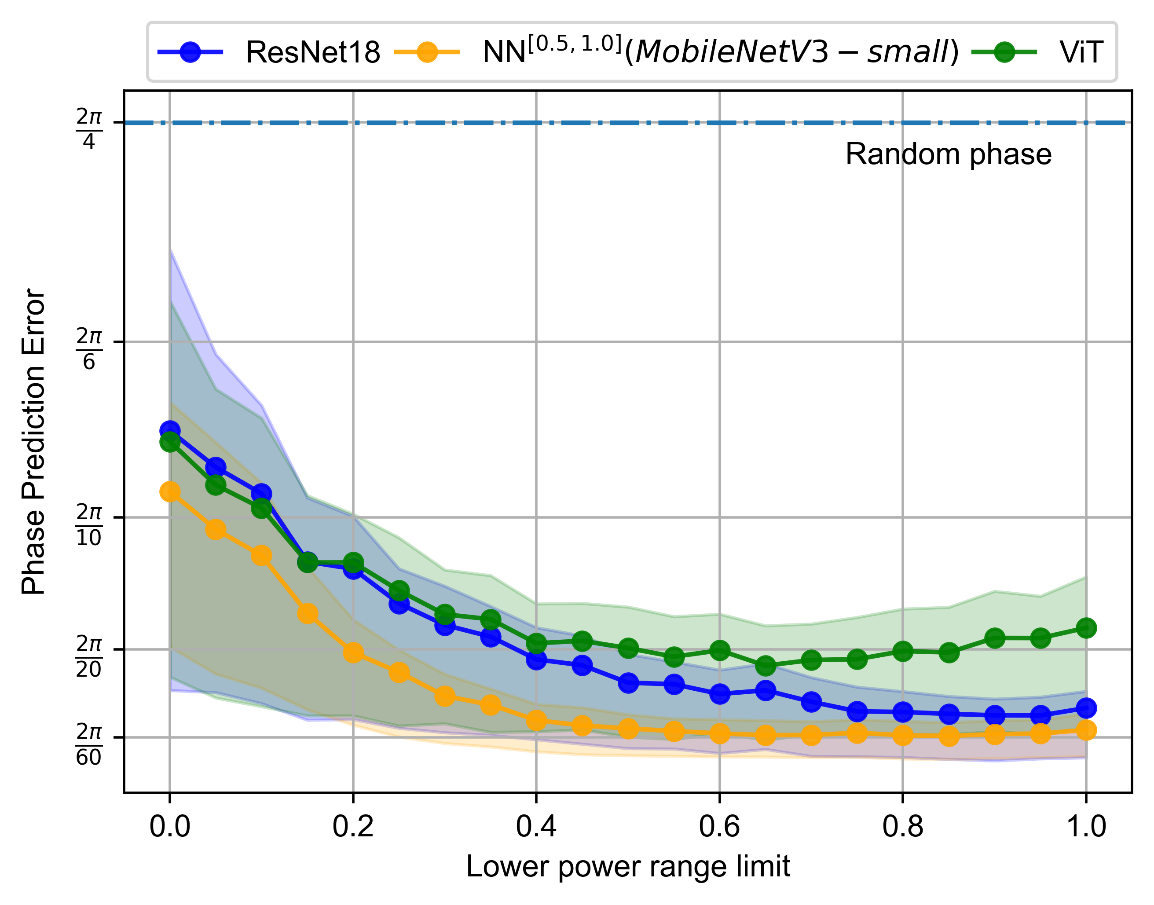


Supplementary Figure 4 Relative performance of ResNet18, ViT and MobileNetV3-Small

As shown in the Supplementary Figure 4, the predictions made by the neural networks exhibit similar trends. Whilst the prediction errors of ResNet-18 and ViT are slightly higher than those of MobileNetV3 (though comparable within the margin of error), these discrepancies could potentially be reduced through hyperparameter optimisation or by adopting larger architectures, such as ResNet-50. Similarly, ViTs can also be designed with greater complexity, such as increasing the number of layers or attention heads, which can provide better performance at the cost of increased computational demands.

However, an additional critical consideration arises that is the latency of the neural network. In this work, the CNN is envisioned as a tool for CBC systems with real amplifiers and phase noise drifts. For instance, ytterbium fibre amplifiers with low differential phase noise above 100 Hz have been demonstrated [3], suggesting that the control algorithm should be operated at operational frequencies that exceed several hundred Hz. To evaluate this requirement, the latencies of ResNet-18, MobileNetV3-Small, and ViT were compared.

The results revealed significant differences in processing frequencies: ResNet-18 achieved an operational frequency of 59.36 ± 13.53Hz, whereas ViT achieved an operational frequency of 133.33 ± 49.78Hz. MobileNetV3-Small, on the other hand, reached 417.88 ± 88.56Hz, showing superior control bandwidth with comparable performance and thus justifying its adoption in this work. These measurements were conducted on a Windows 10 system equipped with an Intel i7-7700 CPU @ 3.60 GHz and an NVIDIA Quadro P6000 GPU.

## Error analysis

To delve deeper into the origins of phase prediction errors, we scrutinised the performance of the neural network denoted as NN^[0.5,1.0]^ in the main manuscript. Supplementary Figure 5 showcases four phase prediction plots generated by NN^[0.5,1.0]^, evaluated on the dataset from Figure 4 of the main text, specifically at datapoints 0.2, 0.4, 0.6, and 0.8. The results reveal a good precision for datapoints 0.6 and 0.8, where nearly all predictions align tightly along the y=x line, reflecting near-ideal accuracy. This excellence is not surprising, as these datapoints fall within the training domain of NN^[0.5,1.0]^, allowing the NN to finely tune its phase extraction for these regions. In contrast, predictions at 0.4 exhibit a modestly wider spread, with more points deviating from the ideal line, and this broadening intensifies at 0.2, where even greater dispersion is evident. Across all cases, however, a recurring pattern emerges: a subset of erroneous predictions clusters conspicuously near ±π.

This concentration can be traced to two factors. First, the cyclic essence of phase poses a challenge, as the network grapples with resolving ambiguities at the boundaries of ±π. The loss function, $arctan2(sin(\theta),cos(\theta))$, confines phases to $[-\pi,\pi]$, yet near these limits, subtle shifts in the true phase can blur the distinction between −π and π, sowing seeds of uncertainty. Second, an intuitive perspective arises from considering two-beam interference, where intensity follows $I=I_{1}+I_{2}+2\sqrt{I_{1}I_{2}}cos(\phi+\delta\phi)$, where $I$ is total intensity, $I_{1}$ and $I_{2}$ are each beam’s intensity, $\phi$ is the reference (i.e., ground truth) phase, and $\delta\phi\ll\phi$ is the phase error. Here, the gradient of the total intensity with respect to phase error flattens near $\phi\approx\pm\pi$, diminishing sensitivity to phase variations. Given that at the observation point, the total interference patterns are dominated by contributions from adjacent pairs, this reduced gradient described by a simple model offers a complementary rationale for the observed degradation near ±π, reinforcing the boundary effect. Together, these insights illuminate the struggles of the NN at these extremes, providing a foundation for understanding and addressing prediction errors.


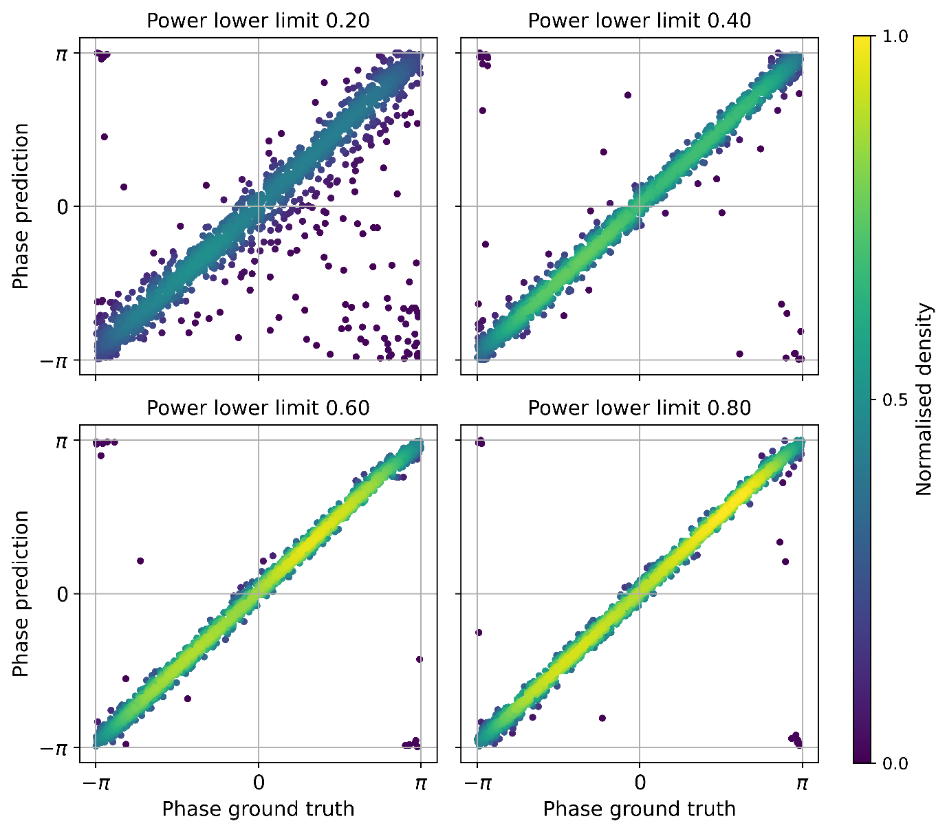


Supplementary Figure 5. Phase prediction accuracies of NN^[0.5,1.0]^ when evaluated on datasets with power ranges [0.20,1.00]‑top left, [0.40,1.00]‑top right, [0.60,1.00]‑bottom left, and [0.80,1.00]‑bottom right.

## Linearity of scalability

A widely used algorithm for phase locking in CBC systems is the Stochastic Parallel Gradient Descent (SPGD)[4]. This iterative algorithm optimises the system performance metric $J=J(\boldsymbol{u})$, where $J(\boldsymbol{u})$ is a function of the control parameters $\boldsymbol{u}=\{u_{1}\boldsymbol{,}\ldots\boldsymbol{,}u_{N}\}$. In CBC systems, the performance metric is typically evaluated by measuring the power on a photoelectric detector with a pinhole placed at the focal plane. The objective is to optimise the control parameters $\boldsymbol{u}$ to maximise $J(\boldsymbol{u})$, thereby achieving "in-phase" locking, where most of the power is concentrated in the central spot.

However, the convergence rate of SPGD degrades roughly as $O\left( N^{2} \right)$, as the number of channels $N$ (i.e., control parameters) increases. In contrast, our studies have demonstrated that NN-based approaches exhibit linear scalability with respect to the number of channels. This difference in scalability can be intuitively understood by examining the regions along the axis of propagation where observations are made for phase-locking algorithms.

For tiled-aperture configurations, three distinct regions can be identified along the axis of propagation. Near the fibre facet**,** there is no overlap between the individual beamlets, and their contributions to the combined intensity pattern are spatially separated. At the focal plane**,** the fields of all beamlets overlap fully. This results in a highly complex interference pattern, where the contributions of individual beamlets could be indistinguishable due to ambiguity, and thus often require iterative phase retrieval methods. Intermediate region (between the fibre facet and the focal plane), in this region, all beamlets partially overlap with their neighbouring beamlets but do not interact significantly with beamlets from afar. Whilst the primary motivation for selecting the intermediate region was to break the Fourier symmetry associated with the focal plane, we also hypothesise that it facilitates the separation of regions of influence for individual beamlets on the combined intensity pattern. This separation, we believe, contributes to the improved scalability and robustness of NN-based approaches compared to traditional SPGD methods.

To support the statement above, we applied the Integrated Gradients (IG) approach[5], which generates “attribution maps” indicating how each input pixel influences the model’s output. Supplementary Figure 6 illustrates CBC system with a 19-beamlet array, where each fibre is numbered. We applied IG to 1,000 Camera A observations using the pretrained NN^[0.5,1.0]^ for the 19-beamlet case depicted in Figure 6 of the main manuscript. This produced 1,000 attribution maps per target, which were then averaged to highlight the most influential regions of the image for each prediction.

Supplementary Figure 7 presents these averaged IG maps for each beamlet’s phase prediction, excluding fibre 9 (which served as a fixed reference and was not predicted). The results show that the most influential pixels, those with higher IG attributions, are localised in the area near each fibre’s initial position, indicating that the neural network primarily focuses on the regions corresponding to each beamlet. This observation is consistent with the previously demonstrated linear scalability in the system’s performance.


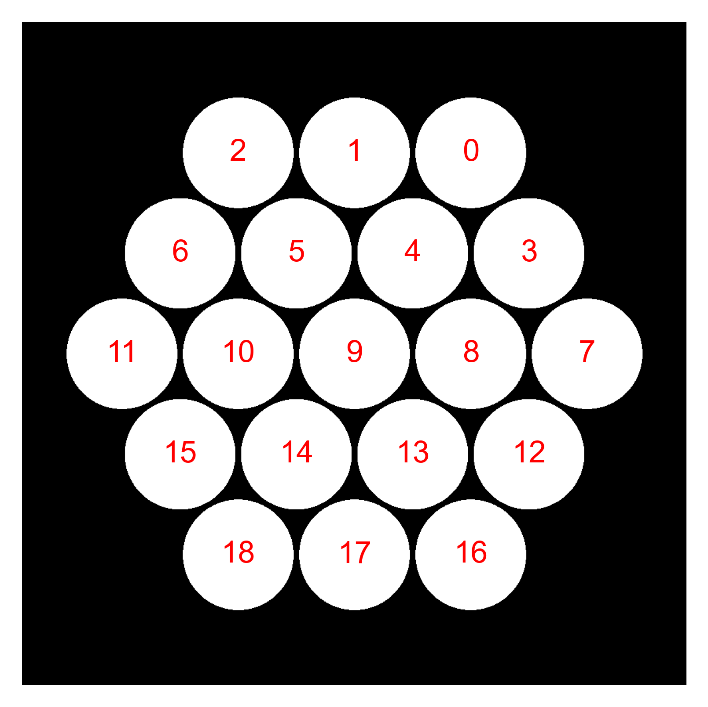


Supplementary Figure 6. 19 fibre CBC hexagonal closed-packed array and the relative position of each respective beamlet.


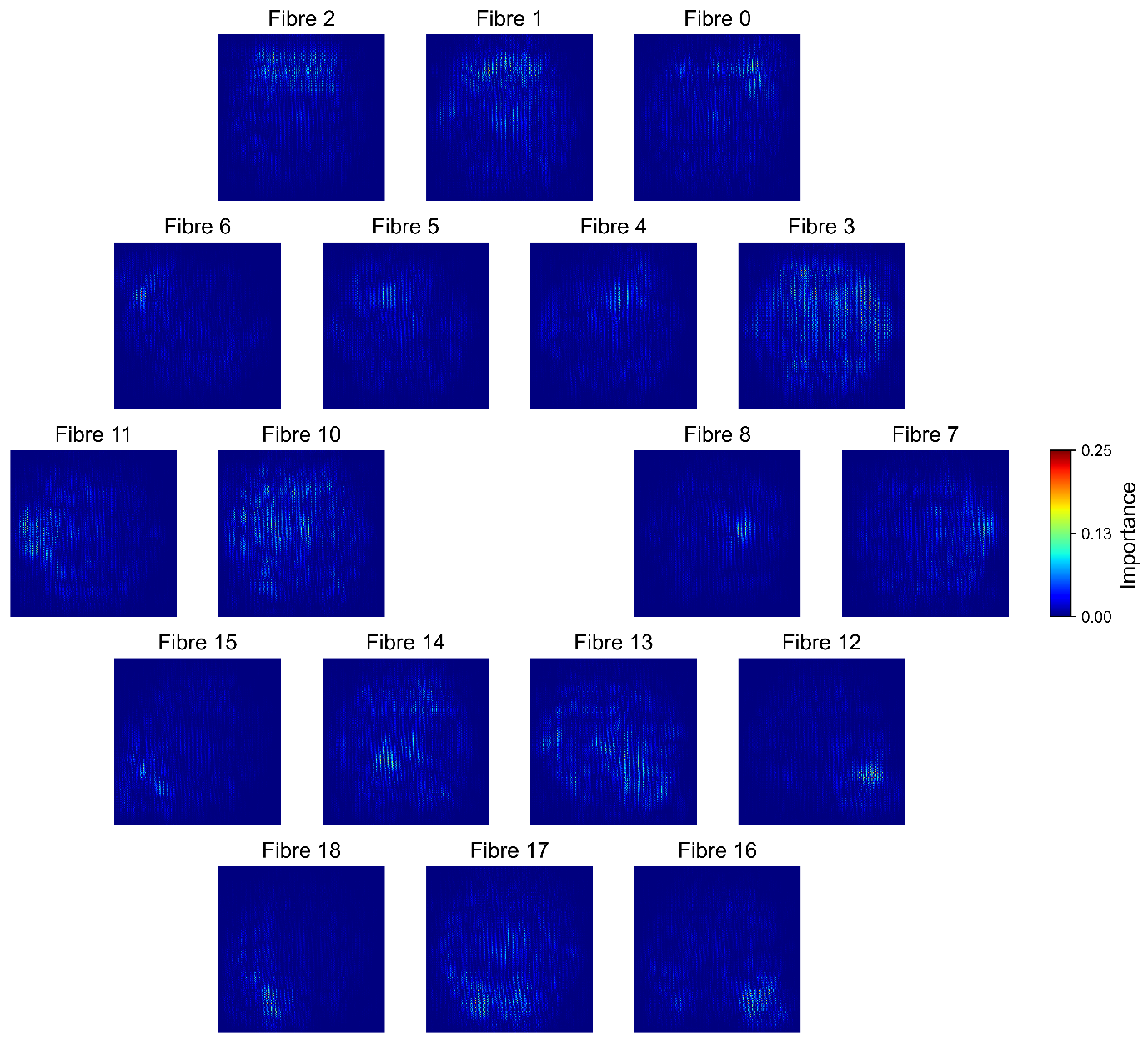


Supplementary Figure 7. Integrated Gradients maps for each beamlet. The numbers in the titles of the gradient maps correspond to the beamlet numbering defined in Supplementary Figure 6. The spatial arrangement of the gradient maps in this figure mirrors the layout of the beamlets in Supplementary Figure 6

To further demonstrate the linearity of our approach, we increased the number of beamlets from 19 to 61. Because a larger beamlet array requires more space on the SLM, the beamlet radii was reduced from 70 to 50 SLM pixels. In addition, the period of the binary grating was changed from 22 to 30 SLM pixels. These modifications ensured that all beamlets fitted onto the SLM panel and that the separation between diffraction orders was sufficient to avoid interference with higher-order diffractions.

New datasets were collected for 3, 7, 19, 37, and 61-beamlet CBC arrays. Similarly, the number of training pairs was gradually reduced and the average phase prediction error was plotted against the number of training pairs, as shown in Supplementary Figure 8a. Next, the number of training pairs required to achieve a phase prediction error in the range $[\frac{0,2\pi}{30}]$ was plotted against the number of beamlets (Supplementary Figure 8b). The higher average phase prediction error, compared with previous results, can be attributed to the adjusted parameters, which were not optimised. As before, both a linear ($k_{1}\cdot N+c_{1}$) and a quadratic ($k_{2}\cdot\frac{N\cdot(N-1)}{2}+c_{2}$) fit were performed on all data points except for the last one, and the results were plotted on the same graph. Notably, the data point for 61 beamlets lies closer to the linear fit, once again corroborating our previous findings.

**
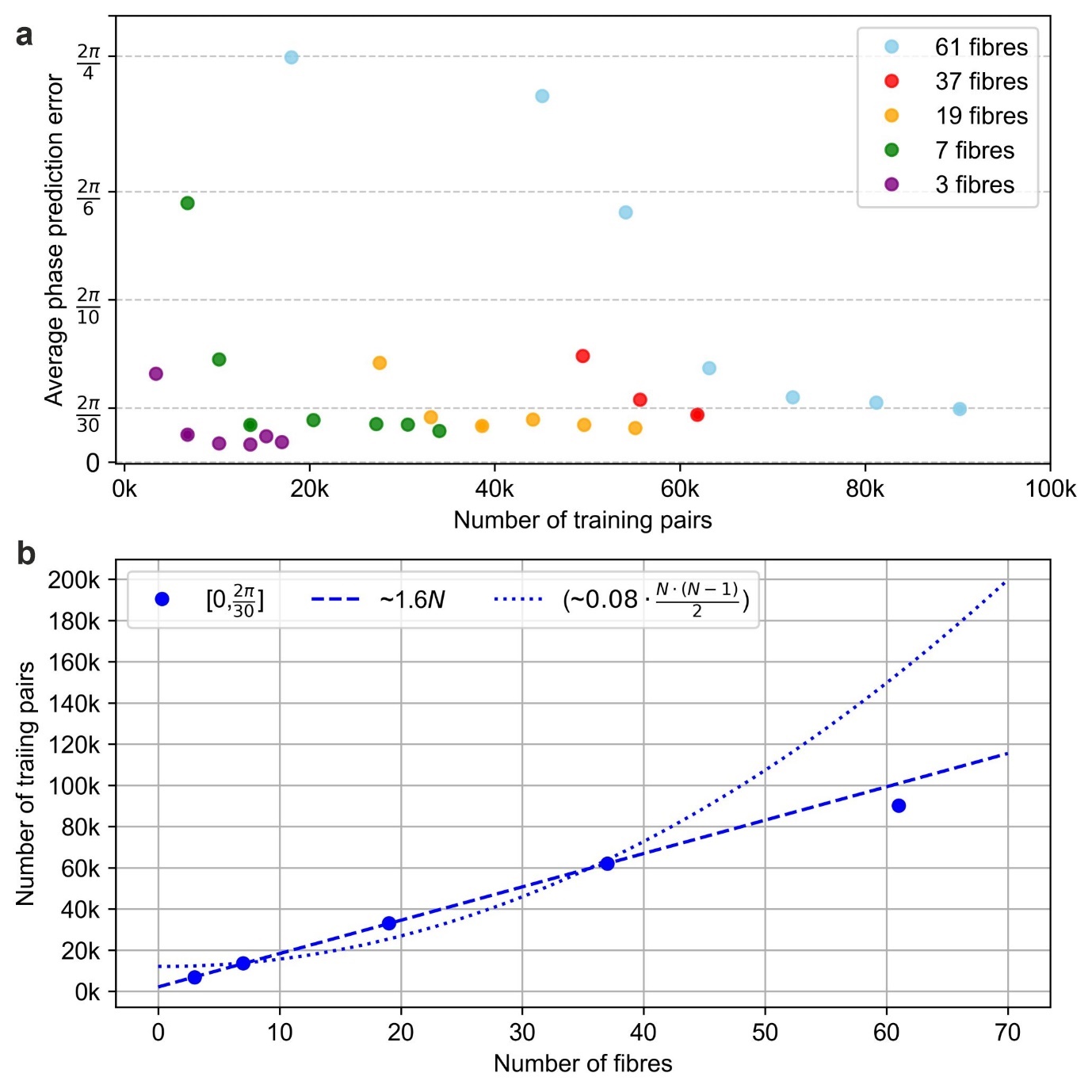
**

Supplementary Figure 8. a) Datapoints illustrating the average phase prediction error after the final epoch of NN^[0.5,1.0]^ training, plotted as a function of the number of training pairs for different CBC arrays., b) Minimum number of training pairs required to achieve phase precision within the range $[0,\frac{2\pi}{30}]$ as a function of the number of fibres. The dashed line represents a linear fit, whilst the dotted line corresponds to the $k_{2}\cdot N\cdot(N-1)/2$ fit.

## Reference

1. Y. Xie, et al., "Single-step phase identification and phase locking for coherent beam combination using deep learning," Scientific Reports **14**, 7501 (2024).

2. A. Dosovitskiy, et al., "An Image is Worth 16x16 Words: Transformers for Image Recognition at Scale," ArXiv **abs/2010.11929**(2020).

3. W. Kerridge-Johns, et al., "Neural Network Coherent Beam Combination with Low Differential Phase Noise Ytterbium Fiber Amplifiers," in *Laser Congress 2024 (ASSL, LAC, LS&C)*, Technical Digest Series (Optica Publishing Group, 2024), ATh1A.6.

4. M. A. Vorontsov and V. P. Sivokon, "Stochastic parallel-gradient-descent technique for high-resolution wave-front phase-distortion correction," J. Opt. Soc. Am. A **15**, 2745-2758 (1998).

5. S. K. Zhongang Qi, Li Fuxin, "Visualizing Deep Networks by Optimizing with Integrated Gradients," (2019 ).
